# Analysis of SARS-CoV-2 mutations in the United States suggests presence of four substrains and novel variants

**DOI:** 10.1038/s42003-021-01754-6

**Published:** 2021-02-15

**Authors:** Rui Wang, Jiahui Chen, Kaifu Gao, Yuta Hozumi, Changchuan Yin, Guo-Wei Wei

**Affiliations:** 1 Department of Mathematics, Michigan State University, East Lansing, MI 48824 USA; 2 Department of Mathematics, Statistics, and Computer Science, University of Illinois at Chicago, Chicago, IL 60607 USA; 3 Department of Electrical and Computer Engineering, Michigan State University, East Lansing, MI 48824 USA; 4 Department of Biochemistry and Molecular Biology, Michigan State University, East Lansing, MI 48824 USA

**Keywords:** SARS-CoV-2, Genome informatics, Viral infection

## Abstract

SARS-CoV-2 has been mutating since it was first sequenced in early January 2020. Here, we analyze 45,494 complete SARS-CoV-2 geneome sequences in the world to understand their mutations. Among them, 12,754 sequences are from the United States. Our analysis suggests the presence of four substrains and eleven top mutations in the United States. These eleven top mutations belong to 3 disconnected groups. The first and second groups consisting of 5 and 8 concurrent mutations are prevailing, while the other group with three concurrent mutations gradually fades out. Moreover, we reveal that female immune systems are more active than those of males in responding to SARS-CoV-2 infections. One of the top mutations, 27964C > T-(S24L) on ORF8, has an unusually strong gender dependence. Based on the analysis of all mutations on the spike protein, we uncover that two of four SARS-CoV-2 substrains in the United States become potentially more infectious.

## Abstract (web summary)

Rui Wang et al. report a comprehensive analysis of nearly 13,000 SARS-CoV-2 genome sequences isolated from patients in the United States, comprising more than 7000 single mutations. They show that SARS-CoV-2 genomes cluster into four distinct groups and that two of these groups are potentially more infectious, underlining the urgent need for viral control strategies in the US.

## Introduction

The ongoing global pandemic of coronavirus disease 2019 (COVID-19) caused by severe acute respiratory syndrome coronavirus 2 (SARS-CoV-2) has led to near a million deaths. The United States (US) has over 6,304,181 confirmed cases and 189,790 deceased cases as of 11 September 2020^1^. The rapid spread of COVID-19 gives rise to a question of whether SARS-CoV-2 has become more transmissible or infectious in the US. We analyze 12,754 complete US SARS-CoV-2 geneome sequences deposited in GISAID^2^ to investigate the characteristics of US SARS-CoV-2 strains and understand their ramifications to the US public health.

SARS-CoV-2 has a genetic proofreading mechanism achieved by non-structure protein (NSP) 14 in synergy with NSP10 and NSP12^3,4^. Therefore, SARS-CoV-2 has a higher fidelity in its transcription and replication process than that of other single-stranded RNA viruses. Nonetheless, 7123 single mutations have been detected in the 12,754 US SARS-CoV-2 strains in the past few months with respect to the first SARS-CoV-2 genome collected on 24 December 2019^5,6^. Genome sequencing, SNP calling, and phenotyping provide an efficient means to study the epidemiology of COVID-19^7^ and infer the relationship between SARS-CoV-2 protein structures and COVID-19 pathogenicity^8–10^. Analyzing genome sequencing and single-nucleotide polymorphism (SNP) calling has been a hotspot for a wide variety of epidemiological, clinical, experimental, biophysical, mathematical, and computational studies^7,9–13^.

Viral virulence and infectivity are some of the most important SARS-CoV-2 characteristics and are determined by its molecular structure and function^14–16^. The intrinsic viral infectivity can be measured by virus quantification that counts the number of viruses in a specific volume over a unit time by using either traditional or modern methods^17,18^, including enzyme-linked immunosorbent assay (ELISA) that is based on protein–protein interactions (PPIs), such as antibody-antigen binding events, being counted by chromogenic or fluorescence reporters. Epidemiological and biochemical studies show that the infectivity of different SARS-CoV strains in host cells is proportional to the binding free energy between the spike (S) protein receptor-binding domain (RBD) and angiotensin-converting enzyme 2 (ACE2) expressed by the host cell^18–22^. Mutation-induced protein–protein binding free energy changes (ΔΔG) of S protein and ACE2 complex provides a means to estimate SARS-CoV-2 infectivity. Alternatively, viral virulence can be quantitatively measured by illness, mortality, or pathological lesions^23,24^. However, these measurements cannot be objective due to interference from disease prevention and treatment.

In this work, we analyze the characteristics of SARS-CoV-2 substrains and 11 prevalent missense mutations in the United States using SNP calling^9,10^, biophysics^25,26^, flexibility-rigidity index (FRI)^25,26^, artificial intelligence^27–29^, algebraic topology^27,30^, and various network theories^11,31^. Our results reveal the following. First, the US SARS-CoV-2 has developed into four substrains. Additionally, three concurrent missense mutations 17747C>T-(P504L), 17858A>G-(Y541C), and 28144T>C-(L84S) tend to fade out, while the other eight concurrent mutations may enhance the infectivity of SARS-CoV-2. Moreover, a US-unique mutation, 27964C>T-(S24L), shows an interesting female-dominated pattern. Furthermore, the US SARS-CoV-2 strains that have 1059C>T-(T85I), 14408C>T-(P323L), 23403A>G-(D614G), 25563G>T-(Q57H), 28144T>C-(L84S), 28881G>A-(R203K), 28882G>A-(R203K), and 28883G>C-(G204R) mutations may become more infectious in the United States. Finally, mutations 23403A>G-(D614G) and 27964C>T-(S24L) likely strengthen the folding stability of the spike protein and ORF8 protein.

## Results and discussion

### SNP calling analysis

#### Cluster analysis

**Fig. 1:**
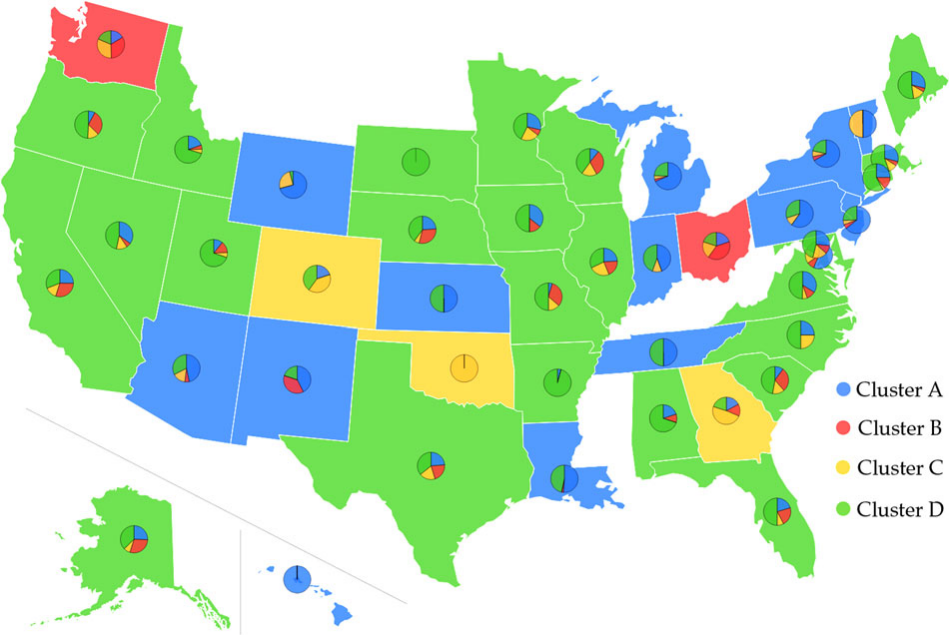
Pie chart plot of four clusters in the United States as of 11 September 2020. The blue, red, yellow, and green colors represent clusters A, B, C, and D, respectively. The base color of each state is decided by its dominant cluster. No color is assigned to a state if we cannot find its complete genome sequences at the GISAID database.

Complete genome sequence data provide us with a wide variety of opportunities to decode the mutation-induced transmission and infection behavior of COVID-19. In this work, we downloaded 45,494 complete SARS-CoV-2 genome sequences from GISAID (https://www.gisaid.org/) up to 11 September 2020. Figure S1 illustrates the distribution of mutations on the SARS-CoV-2 coding genome. A website, called Mutation Tracker, has been created to report the unique single mutations as well as associated frequencies and download related information. Among 45,494 sequences, 12,754 sequences are decoded from the genome isolates submitted by the United States, and 7123 unique single mutations are detected. We track the geographical distributions of the 12,754 US isolates with the *k*-means clustering method (see Fig. S2), showing that the United States are clustered into four distinct clusters, as shown in Fig. 1. The blue, red, yellow, and green represent Cluster A, B, C, and D, respectively. The base color of each state is determined by its dominated cluster. Most of the states are dominated by Cluster A and Cluster D. Table S1 lists the distribution of samples and the total number of single mutations in 20 US states that have submitted many SARS-CoV-2 genome isolates. They are Alaska (AK), Arizona (AZ), California (CA), Connecticut (CT), Washington, D.C. (DC), Florida(FL), Louisiana (LA), Maine (ME), Maryland (MD), Michigan (MI), Minnesota (MN), Nevada (NV), New Mexico (NM), New York (NY), Oregon (OR), Texas (TX), Utah (UT), Virginia (VA), Washington (WA), and Wisconsin (WI). In Cluster A, B, C, and D, the co-mutations with the highest number of descendants are [241C>T, 3037C>T, 14408C>T, 23403A>G], [3037C>T, 14408C>T], [8782C>T, 18060C>T, 28144T>C], and [3037C>T, 14408C>T, 23403A>G] respectively. It is noted that all of the 20 states have the mutations from Clusters A, B, and D. New Mexico (NM) does not have mutations from Cluster C. More analysis related to the infectivity of SARS-CoV-2 based on our four distinct clusters is given in Section Analysis of binding free energy changes.

#### Top mutations in the United States

Here, we focus on the high-frequency or top mutations that represent the most common characteristics of SARS-CoV-2 in the United States. A total of 14 mutations in the United States has a frequency >1000. Among them, 3 mutations are synonymous ones (i.e., 3037C>T-(F106F), 8782C>T-(S76S), and 18060C>T-(L7L)) and 11 mutations are the missense mutations (i.e., 1059C>T-(T85I), 14408C>T-(P323L), 23403A>G-(D614G), 25563G>T-(Q57H), 28144T>C-(L84S), 17858A>G-(Y541C), 17747C>T-(P504L), 27964C>T-(S24L), 28881G>A-(R203K), 28882G>A-(R203K), and 28883G>C-(G204R)). Since synonymous mutations do not change SARS-CoV-2 proteins, we only focus on the other 11 missense mutations. Table 1 lists the frequencies of the top 11 missense mutations in the United States. The dates that these mutations were first detected in the world and in the United States are also included in the Table 1. The first missense mutation with the highest frequency, 23403A>G-(D614G), occurred in China on 24 January 2020. The missense mutation with the second-highest frequency, 14408C>T-(P323L), occurred in Spain on 25 January 2020. Both mutations were first detected in the US on 28 February 2020. The first top mutation recorded in the US was 28144T>C-(L84S), on 19 January. This mutation was originally detected in China on 5 January 2020.

**Table 1:** Top 11 missense mutations that are prevalent in the United States.

Rank: US/World	Mutation	Protein	NC_U.S._	NC_World_	Country	Date
Top 1/Top 1	23403A>G-(D614G)	Spike	10,496	37,294	CN	2020-01-24
					US	2020-02-28
Top 2/Top 2	14408C>T-(P323L)	NSP12(RdRp)	10,491	37,276	ES	2020-01-25
					US	2020-02-28
Top 3/Top 6	25563G>T-(Q57H)	ORF3a	7106	12,323	SG	2020-02-16
					US	2020-02-29
Top 4/Top 7	1059C>T-(T85I)	NSP2	6195	9322	SG	2020-02-16
					US	2020-02-29
Top 5/Top 3	28881G>A-(R203K)	Nucleocapsid	1825	15,837	AU	2020-01-27
					US	2020-02-28
Top 6/Top 4	28883G>C-(G204R)	Nucleocapsid	1824	15,798	AU	2020-01-27
					US	2020-02-28
Top 7/Top 5	28882G>A-(R203K)	Nucleocapsid	1823	15,797	AU	2020-01-27
					US	2020-02-28
Top 8/Top 11	28144T>C-(L84S)	ORF8	1727	3066	CN	2020-01-05
					US	2020-01-19
Top 9/Top 14	17858A>G-(Y541C)	NSP13(Helicase)	1478	1705	US	2020-02-20
					US	2020-02-20
Top 10/Top 15	17747C>T-(P504L)	NSP13(Helicase)	1439	1655	US	2020-02-20
					US	2020-02-20
Top 11/Top 16	27964C>T-(S24L)	ORF8	1176	1248	US	2020-03-09
					US	2020-03-09

The ranking of these 11 mutations in the US and the world are included in the table. NC_U.S._ and NC_World_ represent for the total number of sequences with a specific mutation in the United States and in the world, respectively. The last column records the date that these eight missense mutations were detected for the first time in the world and in the United States. The second-last column records their corresponding countries, i.e., the country lists at the top shows where the mutations first detected, and the country lists at the bottom will always be the United States. Here, AU, US, CN, ES, and SG represent the Australia, the United States, China, Spain, and Singapore, respectively. We use ISO 8601 format YYYY-MM-DD as the date format.

Three of the top 11 missense mutations, i.e., 17858A>G-(Y541C), 17747C>T-(P504L), and 27964C>T-(S24L), appeared in the United States first. In fact, over 87% of these mutations are kept in the United States. Although 28881G>A-(R203K), 28882G>A-(R203K), and 28883G>C-(G204R) have their frequencies being higher than 1000 in the United States, no more than 12% of these three mutations are prevalent in the US. The rest of 88% of 28881G>A-(R203K), 28882G>A(R203K), and 28883G>C-(G204R) are dominated in the European countries. The top mutations in the United States are also the top 7 mutations in the world. However, only one of the next 4 top mutations in the US is ranked within the top 11 globally.

#### Co-mutation analysis

The statistical values of pairwise co-mutations from the top 11 high-frequency mutations in Table 2. The upper triangular reveals the total number of co-mutations for each pair of mutations, the diagonal presents the frequency of every single mutation, and the lower triangular shows the ratios of pairwise co-mutations over single mutations. It is easy to see that the top 11 mutations can be grouped into three essentially disconnected groups. The first group involves 5 mutations together: 1059C>T-(T85I), 14408C>T-(P323L), 23403A>G-(D614G), 25563G>T-(Q57H), and 27964C>T-(S24L) that are strongly correlated, though have a wide range of frequencies. The co-mutations of the second groups have eight unique single mutations: 1059C>T-(T85I), 14408C>T-(P323L), 23403A>G-(D614G), 25563G>T-(Q57H), and 27964C>T-(S24L), 28881G>A-(R203K), 28882G>A-(R203K), and 28883G>C-(G204R). The other three mutations, 17747C>T-(P504L), 17858A>G-(Y541C), and 28144T>C-(L84S), occur mostly together and have similar numbers of frequencies.

**Table 2:** The statistical values of pairwise co-mutations from the top 11 high-frequency mutations.

	1059C>T	14408C>T	23403A>G	25563G>T	27964C>T	28881G>A	28882G>A	28883G>C	17747C>T	17858A>G	28144T>C
1059C>T	6195	6190	6188	6190	1171	8	6	6	0	1	4
14408C>T	0.59/1.00	10,491	10,472	7092	1175	1816	1815	1816	14	5	8
23403A>G	0.59/1.00	1.00/1.00	10,496	7097	1175	1822	1821	1822	13	6	8
25563G>T	0.87/1.00	1.00/0.68	1.00/0.68	7106	1172	10	7	7	1	2	4
27964C>T	1.00/0.19	1.00/0.11	1.00/0.11	1.0/0.16	1176	1	0	0	1	1	1
28881G>A	0/0	1.00/0.17	1.00/0.17	0.01/0.00	0/0	1825	1821	1821	12	3	3
28882G>A	0/0	1.00/0.17	1.00/0.17	0/0	0/0	1.00/1.00	1823	1823	11	2	2
28883G>C	0/0	1.00/0.17	1.00/0.17	0/0	0/0	1.00/1.00	1.00/1.00	1824	11	2	2
17747C>T	0/0	0.01/0.0	0.01/0.00	0/0	0/0	0.01/0.01	0.01/0.01	0.01/0.01	1439	1427	1426
17858A>G	0/0	0/0	0/0	0/0	0/0	0/0	0/0	0/0	0.97/0.99	1478	1472
28144T>C	0/0	0/0	0/0	0/0	0/0	0/0	0/0	0/0	0.83/0.99	0.85/1.00	1727

The values in the diagonal reveal the total number of a specific single mutation in the United States, the values in the upper triangular represent the total number of the co-mutations, and the values in the lower triangular present the ratios of pairwise co-mutations over single mutations.

#### Evolutionary analysis

**Fig. 2:**
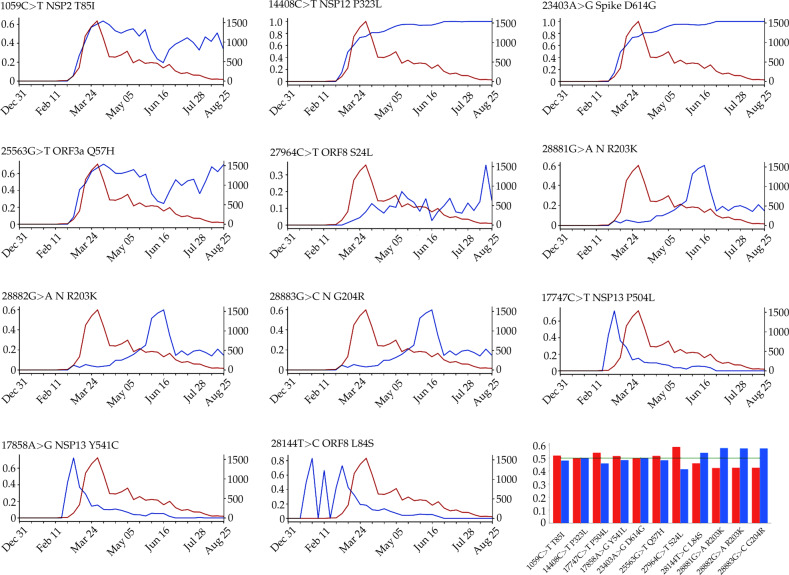
The evolution and the gender distribution of the top 11 missense mutation ratios. The blue lines illustrate the evolution of the top 11 missense mutation ratios (the *y*-axis on the left) computed as the number of genome sequences having a given mutation over the total number of genome sequences. The red lines represent the evolution of the total number of genome sequences (the *y*-axis on the right). The bar plot is the gender distribution of the ratio of the number of samples having top 11 missense mutations over the total number of samples having age and/or gender labels. Red bars represent the female ratios and the blue bars represent the male ratios in the United States.

Figure 2 plots time evolution trajectories of top 11 missense mutations. The red curves are the total numbers of genome samples over time, which become very insufficient after mid May 2020. First, as shown in Table 2, mutations 14408C>T-(P323L) and 23403A>G-(D614G) appear concurrently and thus have an identical trajectory as shown in Fig. 2. This pair of mutations exists in essentially all of the US infections. Additionally, mutation 1059C>T-(T85I) always occurs together with mutation 25563G>T-(Q57H). Therefore, its time evolution trajectory is extremely similar to that of 25563G>T-(Q57H). Both mutations were first detected in Singapore on 16 February 2020. This pair of mutations occur in ~70% of US COVID-19 cases. The third pair of mutations, 17747C>T-(P504L) and 17858A>G-(Y541C), first detected and occurred mostly in the US, have an identical evolution trajectory. Suggested by genome samples, this pair of US-based mutations on the helicase protein appears to have essentially died out in the US. Unfortunately, because there are very insufficient sequencing in the US now as shown by the red curve in Fig. 2, one cannot rule out the existence of these mutations. Mutation 28144T>C-(L84S), the first known mutation globally, has had a very unsteady trajectory. However, its trajectory became identical to those of its co-mutations 17747C>T-(P504L) and 17858A>G-(Y541C) after 20 February 2020. Finally, mutation 27964C>T-(S24L) has an unusual behavior. Its peak ratios occurred in early June when there were insufficient sequence samples in the US.

#### Gender analysis

The last chart in Fig. 2 displays the gender disparity of the top 11 mutations in the US. The overall pattern may correlate with the disparity in male and female immune response and gene editing strengths. In the US, there is an apparent gender difference in mutation 27964C>T-(S24L) on the ORF8 protein. Here, the Fisher’s exact test is applied to verify our deduction that 27964C>T-(S24L) is a female-dominated mutation. Among 1176 samples having 27964C>T-(S24L) mutation, 251 isolates have gender labels (female: 147, male: 104). And 2481 samples do not have the 27964C>T-(S24L) mutation (female: 1201, male: 1280). The *p*-value from the Fisher’s exact test is 0.0028, which is smaller than the significance threshold. Therefore, we can say that mutation 27964C>T-(S24L) has a female-dominance pattern. Actually, C>T mutations also have a female preference^6^, which may be due to the strong host cell mRNA editing known as APOBEC cytidine deaminase^32^.

#### Mutations on 5′UTR

The 5′untranslated region (5′UTR) is a leader RNA fragment, which plays critical roles in the regulation of translation and the gene expression of virus^33,34^. Among 12,754 complete genome sequences in the US, 9673 sequences have mutations on 5′UTR, and there are eight mutations on 5′UTR with frequency >10 in the US. Mutation 241C>T is the most common mutation that has 9628 and 36,786 frequencies respectively in the US and the World, indicating the 241C>T mutation is important for the genomic replication process^33,34^. Mutation 187A>G has the second-highest frequencies in the US and the World, which are 36 and 207, respectively. The frequencies other six mutations (i.e., 199G>T, 222C>T, 208G>T, 218C>T, 242G>T, and 169A>G) are <50. The detailed information can be found in the Supplementary information Table S2.

### Protein-specific analysis

In this section, we discuss the properties of the top 11 missense mutations associated with seven proteins (i.e., NSP2, NSP12, NSP13, Spike, ORF3a, ORF8, and Nucleocapsid). Figure S3 illustrates the proteoforms of SARS-CoV-2 proteins. Moreover, to understand the impact on the protein’s structures induced by mutations, we employ artificial intelligence^27^, FRI^26^, and subgraph centrality models^11^ as summarized in Table 3.

**Table 3:** The protein folding stability changes of 11 missense mutations.

Rank	Mutation	Protein	ΔΔ*G*(kcal/mol)	${R}_{8}^{w}$	${R}_{8}^{m}$	$\Delta {\bar{R}}_{8}$%	$\langle {C}_{s}^{w}\rangle$	$\langle {C}_{s}^{m}\rangle$	$\Delta \langle {\bar{C}}_{s}\rangle$%
Top 1	23403A>G-(D614G)	S protein	0.34	10.27	10.10	1.7	2376	1386	42
Top 2	14408C>T-(P323L)	NSP12(RdRp)	−0.11	9.77	9.87	−1.0	1105	1959	−77
Top 3	25563G>T-(Q57H)	ORF3a	−0.24	11.33	11.66	−1.5	25,061	58,592	−134
Top 4	1059C>T-(T85I)	NSP2	−0.05	12.37	12.51	−1.1	89,764	166,399	−85
Top 5	28881G>A-(R203K)	Nucleocapsid	−1.14	15.69	15.44	1.6	19,356,251	9,436,565	51
Top 6	28883G>C-(G204R)	Nucleocapsid	−1.56	14.99	16.94	13	1,193,960	1,191,199,736	−99,669
Top 7	28882G>A-(R203K)	Nucleocapsid	−1.14	15.69	15.44	1.6	19,356,251	9,436,565	51
Top 8	28144T>C-(L84S)	ORF8	−0.99	12.28	12.05	1.9	12,810	6504	49
Top 9	17858A>G-(Y541C)	NSP13(Helicase)	−0.81	11.52	10.40	9.7	506,640	7271	99
Top 10	17747C>T-(P504L)	NSP13(Helicase)	−0.59	7.52	7.54	0.3	4668	6094	−31
Top 11	27964C>T-(S24L)	ORF8	0.20	11.72	11.66	0.5	11,685	29,777	−155

The folding stability change ΔΔ*G* = Δ*G*_w_ − Δ*G*_m_, where Δ*G*_w_ and Δ*G*_m_ are the folding free energies of the wild type and the mutant type, respectively. ${R}_{8}^{{\mathrm{w}}}$ and ${R}_{8}^{{\mathrm{m}}}$ are FRI rigidities for the wild type and mutant type of the protein with *η* = 8 Å. Here, $\langle {C}_{s}^{{\mathrm{w}}}\rangle$ and $\langle {C}_{s}^{{\mathrm{m}}}\rangle$ are the average subgraph centralities of the wild type and the mutant type, respectively. $\Delta {\bar{R}}_{8}$ and $\Delta \langle {\bar{C}}_{s}\rangle$ are the molecular FRI rigidity changes and the average subgraph centrality change.

#### Mutation on the NSP12 protein

**Fig. 3:**
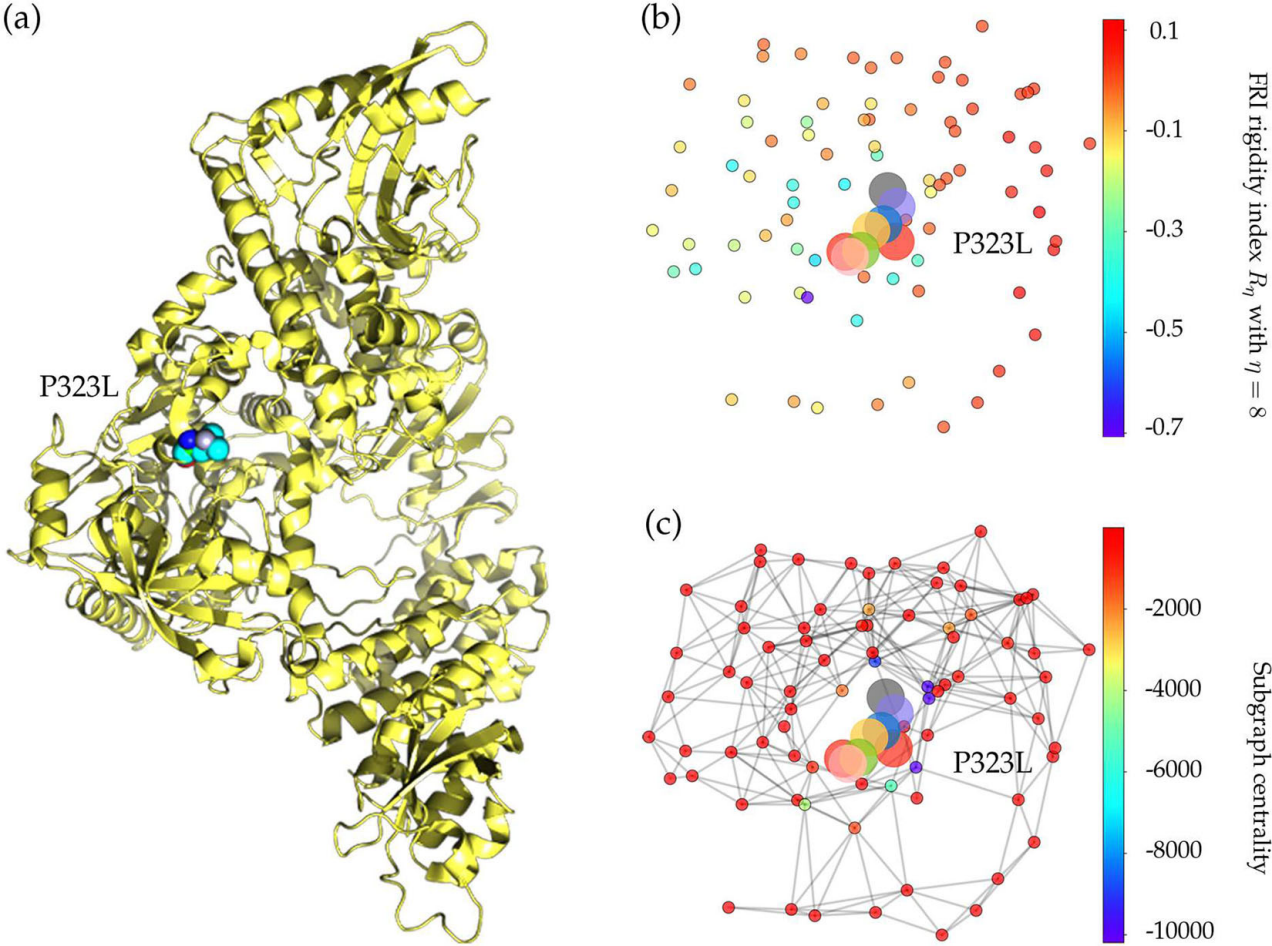
The 3D structure and network analysis of SARS-CoV-2 NSP12 protein. **a** The 3D structure of SARS-CoV-2 NSP12 protein. The mutated residue is marked with color balls. **b** The differences of FRI rigidity index between the network with wild type and the network with mutant type. **c** The difference of the subgraph centrality between the network with wild type and the network with mutant type.

Mutation 14408C>T-(P323L) on the NSP12 (aka RNA-dependent RNA polymerase (RdRp)) is one of dominant mutations in the United States. Among 12,754 complete genome sequences, 5918 are connected to P323L. Figure S4 shows the sequence alignment for the NSP12 of SARS-CoV-2, SARS-CoV^35^, bat coronavirus RaTG13^36^, bat coronavirus CoVZC45^37^, and bat coronavirus BM48-31^38^. The red rectangle marks the mutant residue with its neighborhoods. SARS-CoV-2 NSP12 is highly conservative among the other four species. Although P323L mutates the residue of proline (P) to leucine (L), these two residues are both non-polar and aliphatic, indicating P323L may not affect the functionality of NSP12. Figure 3a shows the three-dimensional (3D) structure of SARS-CoV-2 NSP12 created by PyMol^39^.

NSP12 is of paramount importance to the SARS-CoV-2 replication and transcription machinery^13,40^. The increasing ratio of P323L in Fig. 2 indicates that this type of mutations may favor SARS-CoV-2 and enhance the transmission capacity of SARS-CoV-2. However, a more likely reason is that P323L is a co-mutation of D614G, suggesting that mutation P323L may be enhanced by mutation D614G. The negative folding stability changes in Table 3 suggest that P323L destabilizes the NSP12. Figure 3b, c show the differences of FRI rigidity index and subgraph centrality between the network with wild type and the network with mutant type. The atoms on the mutant residue are mark with big colored balls. We deduce that the slight increase in the rigidity means the mutation makes the protein less flexible or less cooperative in synergistic interactions.

From Table 2, we can see that 14408C>T-(P323L) always shows up with 1059C>T-(T85I), 23403A>G-(D614G), 25563G>T-(Q57H), and 27964C>T-(S24L) simultaneously. Therefore, we can deduce that the increasing tendency of P323L ratios per 7-days is due to its co-mutation with other infectivity-strengthening mutations, such as 23403A>G-(D614G).

#### Mutation on the spike protein

Mutation 23403A>G-(D614G) located on the spike protein has the second-highest frequency in the United States, which has been considered as the key mutation that makes SARS-CoV-2 more infectious worldwide^12,41,42^. From Table 1, one can see that mutation D614G was initially detected in China on 24 January 2020. The first case with the D614G mutation in the United States was reported on 28 February 2020 in Florida (2 sequences) and Rhode Island (1 sequence). A higher prevalence of D614G in the east coast of the US was reported^43^. Since then, SARS-CoV-2 with the mutation D614G has become a major variant, and 82.3% of patients carry D614G in the United States as of 11 September 2020.

**Fig. 4:**
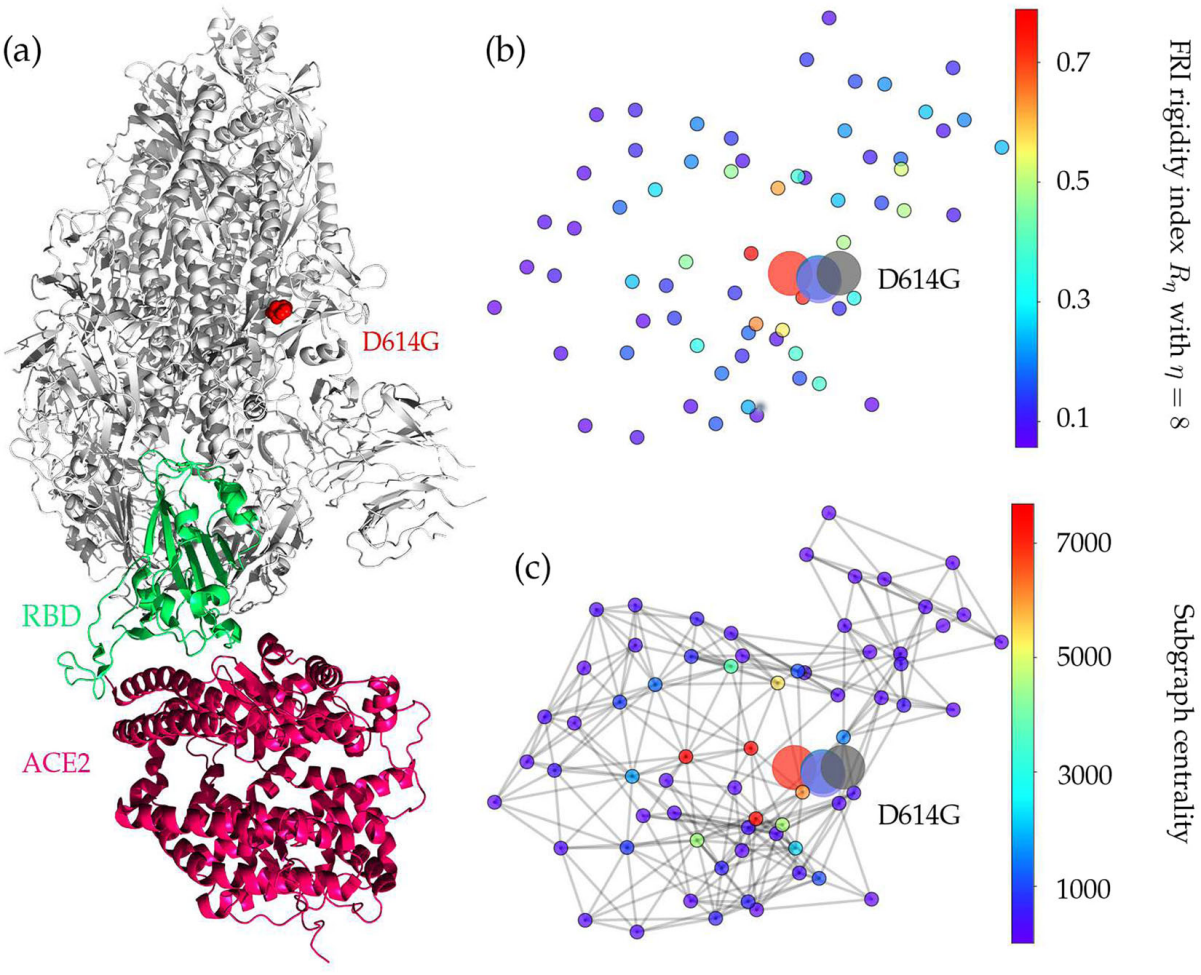
The 3D structure and network analysis plot of SARS-CoV-2 S protein. **a** Illustration of S-protein and ACE2 interaction. The RBD is displayed in green, the ACE2 is given in red, and mutation D614G is highlighted in red. **b** The difference of FRI rigidity index between the network with wild type and the network with mutant type. **c** The difference of the subgraph centrality between the network with wild type and the network with mutant type.

Figure S5 depicts the sequence alignment for the S protein in different species. The S protein of bat coronavirus RaTG13 has the highest similarity of 97.47% with the S protein of SARS-CoV-2. Amino acids near position 614 are very conservative, indicating that D614G mutation will play an important role in the functions of the S protein of SARS-CoV-2. Figure 4a depicts the 3D structure of the SARS-CoV-2 spike protein that interacts with the host ACE2. The D614G mutation is one of the most prevalent mutations of SARS-CoV-2, which changes the amino acid aspartate (D) with the polar negative charged side changes to the amino acid glycine (G) with a non-polar side chain. Moreover, the D614G mutation ratio in Fig. 2 keeps climbing, and the ratio is approaching the unity after 16 June 2020, which also proves that SARS-CoV-2 becomes more contagious as time goes on.

Table 3 shows a positive folding free energy change, indicating the stabilization effect of the mutation. Figure 4b, c illustrate the difference of FRI rigidity index and the subgraph centrality between the network with wild type and the network with mutant type. The FRI rigidity decreases following the mutation, endowing the S protein higher flexibility to interact with ACE2. The same is confirmed by average subgraph centrality.

#### Mutation on the ORF3a protein

**Fig. 5:**
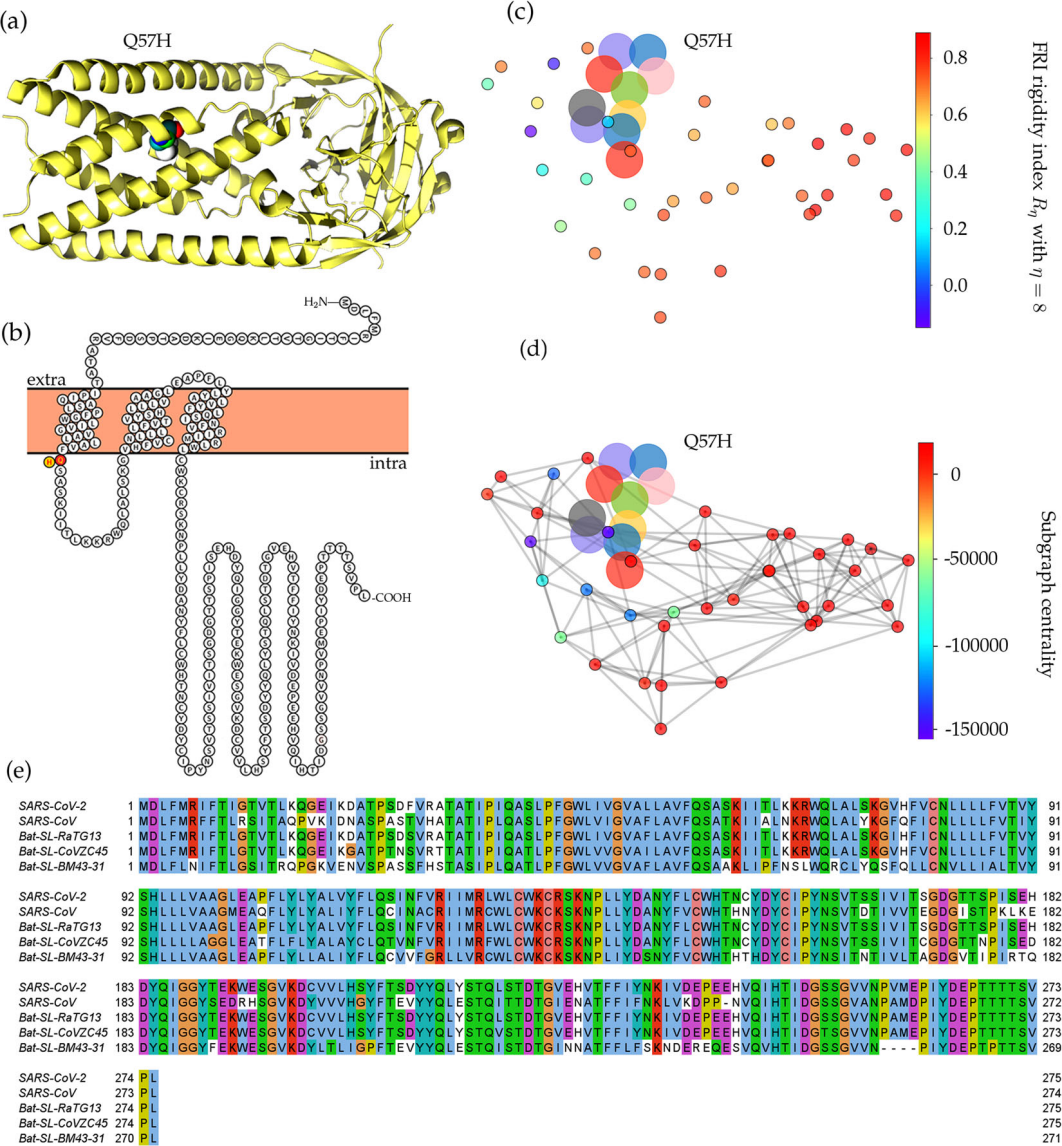
The 3D structure and network analysis plot of SARS-CoV-2 ORF3a protein. **a** The 3D structure of SARS-CoV-2 ORF3a protein. **b** The visualization of SARS-CoV-2 ORF3a proteoform. The high-frequency mutation 25563G>T-(Q57H) on ORF3a is marked in color. The red color represents the wild type and the yellow represents the wild type. **c** The difference of FRI rigidity index between the network with wild type and the network with mutant type. **d** The difference of the subgraph centrality between the network with wild type and the network with mutant type. **e** Sequence alignments for the ORF3a protein of SARS-CoV-2, SARS-CoV, bat coronavirus RaTG13, bat coronavirus CoVZC45, and bat coronavirus BM48-31. Detailed numbering is given according to SARS-CoV-2. One high-frequency mutation 25563G>T-(Q57H) locates on the ORF3a protein. Here, the red rectangle marks the Q57H position with its neighborhoods.

Mutation 25563G>T-(Q57H) is on the ORF3a protein. The Q57H mutation changes the amino acid glutamine (Q) with a non-charged polar side chain to the positively charged polar side chain of amino acid histidine (H). Figure 5e shows the sequence alignment results in different species. The ORF3a of SARS-CoV-2 and SARS-CoV share a 71.53% sequence similarity. The amino acids nearby position 57 are all conservative in all five species. Moreover, Fig. 5b visualizes the SARS-CoV-2 ORF3a proteoform, where we use red to mark the wild-type amino acid glutamine (Q) and yellow to address the mutant amino acid histidine (H). Mutation Q57H locates at the intramolecular interface and in touch with the membrane, which indicates the special functionality changes that Q57H can induce. Figure 5b is the visualization of ORF3a, which is generated by an online server Protter^44^.

SARS-CoV-2 ORF3a protein is widely expressed in intracellular and plasma membranes, which induces apoptosis and inflammatory responses in the infected cells and transfected cells^45,46^. Figure 5c, d depict the differences of the FRI rigidity index and the subgraph centrality between the network with wild type and the network with mutant type. The ORF3a becomes more rigidity after the mutation, which may result in a less flexible mutant for ORF3a to involve in the apoptosis and inflammatory response.

As illustrated in Fig. 2, the ratio of the 25563G>T-(Q57H) mutation on ORF3a in each 7-day period kept increasing once it was introduced to the United States. This tendency indicates that mutation Q57H becomes prevalent in the viral patients of the United States, which may make the SARS-CoV-2 more infectious. From Table 2, we can see that 7106 sequences have 25563G>T-(Q57H) mutation on ORF3a. Among them, 7079 variants have the [25563G>T-(Q57H), 23403A>G-(D614G)] co-mutations. The negative folding stability changes of mutation Q57H in Table 3 reveals that ORF3a becomes unstable following the Q57H mutation, which may harm the function of ORF3a in apoptosis and increase the viral load in the host cell. However, the Q57H mutation locates near TNF receptor-associated factors (TRAFs), ion channel, and caveolin binding domain^46^, which may affect the NLRP3 inflammasome activation. ORF3a activates the innate immune signaling receptor NLRP3 inflammasome and causes tissue inflammation and cytokine production^47^.

#### Mutation on the NSP2 protein

As of 11 September 2020, more than half of mutation 1059C>T-(T85I) counts found worldwide are from the United States. The residue T85 on the NSP2 is polar and non-charged, and it changes to a non-polar residue I85 after the mutation. Figure S6a in the Supplementary Information shows the 3D structure of SARS-CoV-2 NSP2. From Fig. S7, we can see that coronaviral NSP2 is relatively conservative for the first 91 residues. Moreover, the T85 residue with its neighbors is all conservative in the other four SARS-like sequences, indicating this type of mutations may be substantial to coronaviral structures and properties.

NSP2 is also a viral protein that does not attract much research attention. In the SARS-CoV genome, the deletion of NSP2 may only result in a modest reduction in viral titers, which is considered to be a dispensable protein^48^. Table 3 shows that the folding stability change of T85I is −0.05 kcal/mol. Although the negative value reveals that T85I may destabilize the structure of NSP2, this small change is negligible. The FRI rigidity change is also minor, as is shown in Fig. 3c, indicating the mutation of T85I on the NSP2 does not change the flexibility of NSP2 too much. However, the growing trend in Fig. 2 still indicates that 1059C>T-(T85I) is an infectivity-strengthening mutation, which may mainly benefit from the co-mutation with other infectivity-strengthening mutations, such as 23403A>G-(D614G) and 25563G>T-(Q57H).

#### Mutations on the NSP13 protein

NSP13 of SARS-CoV-2 is a superfamily 1 helicase, which can unwind a double-stranded RNA (dsRNA) or DNA (dsDNA) in the 5′ to 3′ direction into two single-stranded nucleic acids^49–51^. As illustrated in Fig. S8, NSP13 of SARS-CoV-2 shares the most homology with the other four species and is one of the most conservative proteins in SARS-CoV-2 genome. Therefore, the existence of two high-frequency mutations on the NSP13 is very unusual. Similar to 27964C>T-(S24L), although 17858A>G-(Y541C), 17747C>T-(P504L) are in the final list in Table 1, more than 87% of them were detected in the United States. Mutation Y541C changes the amino acid tyrosine (Y) to cysteine (C). Figure S9a in the Supplementary Information shows the 3D structure of SARS-CoV-2 NSP13. Notably, tyrosine has an aromatic side chain, while cysteine only has a polar non-charged side chain, indicating that the 3D structure of NSP13 will be incredibly affected.

From Fig. 2, both mutations on the NSP13 have the same trajectory of the mutation ratios over time. Once mutations 17858A>G-(Y541C) and 17747C>T-(P504L) were first found in the United States, they had a rapid increase in the first 2 weeks. However, these two mutations do not benefit SARS-CoV-2. In early March, the ratio of both mutations start to decrease and approach zero after 19 May 2020, suggesting that mutations 17858A>G-(Y541C) and 17747C>T-(P504L) may hinder the transmission of SARS-CoV-2. In Table 2, 17858A>G-(Y541C) and 17747C>T-(P504L) do not show up with 23403A>G-(D614G) in more than a thousand SNP profiles. As reported in Deng et al.^52^, 17858A>G-(Y541C) and 17747C>T-(P504L) occurred among Grand princess strains and some WA state strains (aka WA1 lineage). One possible reason for the declined ratios for 17858A>G-(Y541C) and 17747C>T-(P504L) may be due to the efficient lockdown measures enforced in WA and CA at the early stage of the outbreak in the US. Moreover, another possible reason is that mutations 17858A>G-(Y541C) and 17747C>T-(P504L) may weaken the transmission capacity of SARS-CoV-2. However, we hope that interested labs could verify our assumption through culture/animal studies.

Table 3 shows that both high-frequency mutations Y541C and P504L have negative folding stability changes, which will destabilize the structure of NSP13. Mutations 17858A>G-(Y541C) and 17747C>T-(P504L) happen simultaneously after analyzing 45,494 genome sequences, indicating the folding stability changes on the NSP13 are superimposed by two simultaneously occurred mutations. This also explains the same decreasing tendency in Fig. 2 after early March. Based on the protein-specific analysis mentioned above, we deduce that mutations Y541C and P504L prevent SARS-CoV-2 from efficiently interacting with host interferon signaling molecules and impede the NSP13 from efficacious participation in the replication/transcription process. Figure 3b shows the difference of the FRI rigidity index between the network with wild type and the network with the mutant type. One mutation (17747C>T-(P504L)) does not affect the rigidity much, whereas the other mutation (17858A>G-(Y541C)) leads to a decrease in the NSP12 rigidity, which may make NSP13 not as robust as before to involve in the viral infection and replication process.

#### Mutations on the ORF8 protein

The ORF8 protein has two high-frequency mutations, 28144T>C-(L84S) and 27964C>T-(S24L). More than 94.2% mutation 27964C>T-(S24L) worldwide were found in the United States. The first confirmed case with 27964C>T-(S24L) was discovered on 9 March 2020, in the United States, suggesting that S24L initially happened in the US. Additionally, these two high-frequency mutations S24L and L84S mutate reversibly. The amino acid serine (S) has a non-charged polar side chain, while the leucine (L) has a non-polar aliphatic residue. Figure S10 illustrates the sequence alignment of SARS-CoV-2 ORF8 with the other four species. The SARS-CoV-2 ORF8 shares a really low similarity among all the other four SARS-like species. SARS-CoV, Bat coronavirus RaTG13, Bat coronavirus CoVZC45, and Bat coronavirus BM48-31 have the same residues at positions 24 and 84. Nonetheless, SARS-CoV-2 ORF8 owns different types of residues. Here, we would like to address that although the ORF8 of SARS-CoV-2 at position 84 has a different residue compared to the other four species, it mutates back to S in 1727 isolates in the United States.

Figure S11a in the Supplementary Information shows the 3D structure of SARS-CoV-2 ORF8. ORF8 protein of SARS-CoV-2 shares the least homology with SARS-CoV among all viral proteins, which mediates the immune evasion by interacting with major histocompatibility complex molecules class I (MCH-I) and down-regulating the surface expression of MHC-I on various cells^53,54^. The overall upward trend of the S24L ratio over time reveals that S24L may enhance SARS-CoV-2’s ability to spread. In contrast, the time evolution plot shows that the ratio of mutation 28144T>C-(L84S) goes up before the beginning of March, and then the ratio goes down and approach zero after 23 May 2020. Due to the small number of sequence data, we can say that the ratio of L84S has a decreasing tendency. The female patients with S24L mutation on ORF8 account for a large proportion, which indicates that the S24L is most likely to happen in the female population in the United States.

Table 3 shows that the folding stability change of 28144T>C-(L84S) is −0.99 kcal/mol, indicating that ORF8 becomes unstable. Figure S10b in the Supplementary Information shows that the ORF8 becomes slightly less rigidity after both L84S and S24L mutations. The rigidity changes induced by S24L is less than the L84S. Based on the function of ORF8 that involved in the immune response, we deduce that L84S may be one of the factors that disfavor SARS-CoV-2 and favor the host immune surveillance to decrease the viral load in the human cells, which provides an explanation that the ratio of L84S in Fig. 2 keeps decreasing. Meanwhile, the positive folding stability change of 27964C>T-(S24L) lists in Table 3 reveals that this type of mutation may enhance the function of ORF8. Therefore, the MHC-I will be inhibited more, and the eradication of SARS-CoV-2 in vivo will be hindered. This could be one possible reason why the ratio of S24L is on the rise. Notably, after analyzing 28,726 complete genome sequences, none of them have mutations 28144T>C-(L84S) and 27964C>T-(S24L) happened simultaneously.

#### Mutations on the nucleocapsid protein

Nucleocapsid (N) protein is a structural protein for the SARS-CoV-2, which participates in the RNA packaging, virus particle releasing, and the ribonucleoprotein core forming process^55^. Figure S12 in the Supplementary Information illustrates the 3D structure of SARS-CoV-2 N protein. The high-frequency mutations that detected on the Nucleocapsid (N) protein are 28881G>A, 28881G>A, and 28883G>C. From Fig. 2, we can see that the ratio of these three mutations increase rapidly after early May, and in the middle of June, their ratios start to decline. Only 14.3% sequences in the US have mutations 28881G>A, 28881G>A, and 28883G>C.

R203K is caused by both 28881G>A and 28882G>A. On the protein level, mutation 28883G>C leads to G204R. Figure S13 is the sequence alignments for the N protein of SARS-CoV-2, SARS-CoV, bat coronavirus RaTG13, bat coronavirus CoVZC45, and bat coronavirus BM48-31. We can see that positions 203 and 204 are both conservative. Residues Arginine (R) and Lysine (K) are both positively charged. Therefore, the mutation R203K may not affect N protein function. To be noted, Glycine (G) is a non-polar residue. Mutation 28883G>C-(G204R) may affect the hydrophilicity of N protein, which is in consistent with the predicted negative folding stability changes of R203K and G204 shown in Table 3.

### Analysis of binding free energy changes

SARS-CoV-2 enters the host cell by the interaction of the S protein and ACE2. The viral S protein is primed by TMPRSS2 to entail its cleavage at two potential sites, Arg685/Ser686 and Arg815/Ser816^18^. Epidemiological and biochemical studies show that the infectivity of different SARS-CoV strains in host cells is proportional to the binding free energy between the S protein RBD and host ACE2^18–22^. Therefore, the infectivity of SARS-CoV-2 can be theoretically estimated by the predicted RBD-ACE2 binding free energy. Additionally, since the natural selection favors infectivity-strengthening mutations, those mutations that are predicted to have positive binding free energy changes should be observed more frequently.

We found 434 non-degenerate single mutations on the spike protein. Among them, 46 single mutations are detected on the receptor-binding domain (RBD), and 19 single mutations occurred on the receptor-binding motif (RBM). We separate 12,754 complete SARS-CoV-2 genome sequences in the US into four clusters and calculate the mutation-induced binding free energy changes of S protein RBD and ACE2 in each cluster, which will help us understand the potential transmission tendency induced by the mutations on the S protein RBD. The binding free energy change induced by single mutation ΔΔ*G* = Δ*G*_W_ − Δ*G*_M_ is defined as the subtraction of the binding free energy of the mutant type (Δ*G*_M_) from the binding free energy of the wild type (Δ*G*_*W*_). Furthermore, the positive binding free energy change of a single mutation means that the mutation can enhance the binding free energy of the S protein RBD and ACE2 and make SARS-CoV-2 more infectious.

**Fig. 6:**
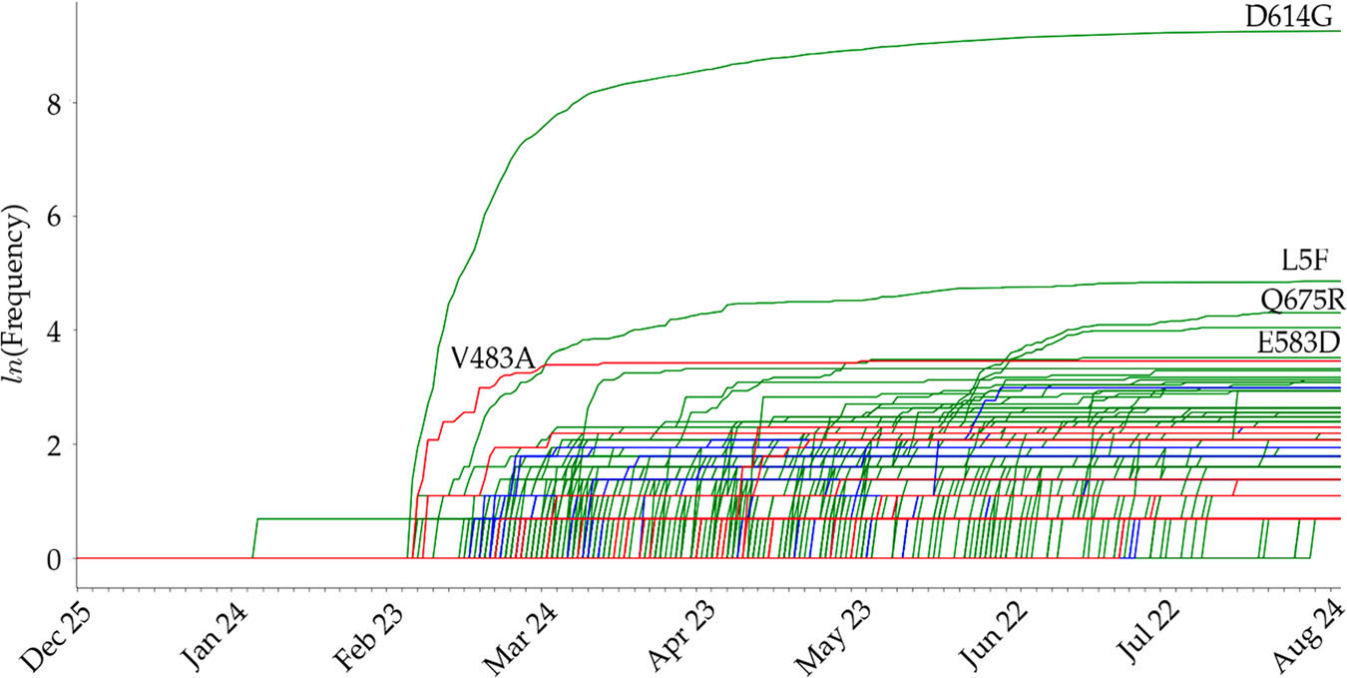
The time evolution of 264 SARS-CoV-2 S protein mutations. The red lines represent the RBD mutations that strengthen the infectivity of SARS-CoV-2 (i.e., ΔΔ*G* is positive), the blue lines represent the RBD mutations that weaken the infectivity of SARS-CoV-2 (i.e., ΔΔ*G* is negative), and the green lines are for S protein mutations that away from the RBD. The mutation with the highest frequency is D614G.

Figure 6 illustrates the time evolution trajectories of 434 single mutations on SARS-CoV-2 S protein RBD. The red line shows the mutations that have positive predicted binding free energy changes and the blue lines represent the mutations that have negative predicted binding free energy changes. The red lines gradually outpace the blue lines as time progresses, suggesting that these mutations are favored by natural selection that may enhance the infectivity of SARS-CoV-2. Additionally, green lines indicate the evolutions of mutations that locate away from the RBD. The mutation that has the highest frequency is D614G, which was reported to enhance SARS-CoV-2 infectivity^56,57^. The trajectories of the other two high-frequency S protein mutations (Q675R and E583D) indicate that they are co-mutations with infectivity-enhancing S protein mutations, such as D614G. We found that the other high-frequency S protein mutation L5F is independent of mutation D614G.

**Fig. 7:**
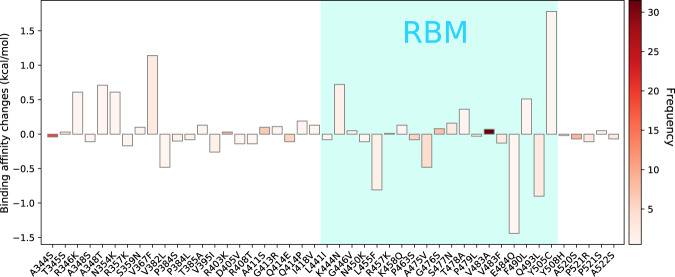
Overall binding free energy changes ΔΔ*G* (kcal/mol) on the receptor-binding domain (RBD). The blue color region marks the binding free energy changes on the receptor-binding motif (RBM). The height of each bar indicates the predicted ΔΔ*G*. The color indicates the occurrence frequency in the GISAID genome dataset.

Figure 7 illustrates overall predicted binding free energy changes ΔΔ*G* (kcal/mol) induced by 46 single mutations on SARS-CoV-2 S protein RBD. The color bar on the left-hand side of the figure represents the mutation frequency. Here, 41% single mutations have positive binding changes (19 out of 46). Moreover, the frequency of mutations with positive predicted binding free energy changes is higher than those with negative predicted binding free energy changes, suggesting that SARS-CoV-2 is more likely to be infectious.

Mutation 23010T>C-(V483A) has the highest frequency (31) localized on the RBM has the positive binding free energy change, which indicates that V483A is prevalent in COVID-19 patients’ in the United States has a potential capacity to enhance the infectivity of SARS-CoV-2. However, mutations that locate away from the RBM will also have a crucial impact on the infectivity^58^. Although away from the RBM, the relatively high-frequency and positive binding free energy changes of V367F, R403K, and A411S indicate that more attention should be paid to them in the future. Additionally, an interesting finding is that the mutations occurred at the same residue position such as A348S and A348T, P384L and P384S have similar binding free energy changes. Based on the SNP calling results, we separate 12,754 SNP variants from the United States into four clusters.

#### Cluster A infectivity

**Fig. 8:**
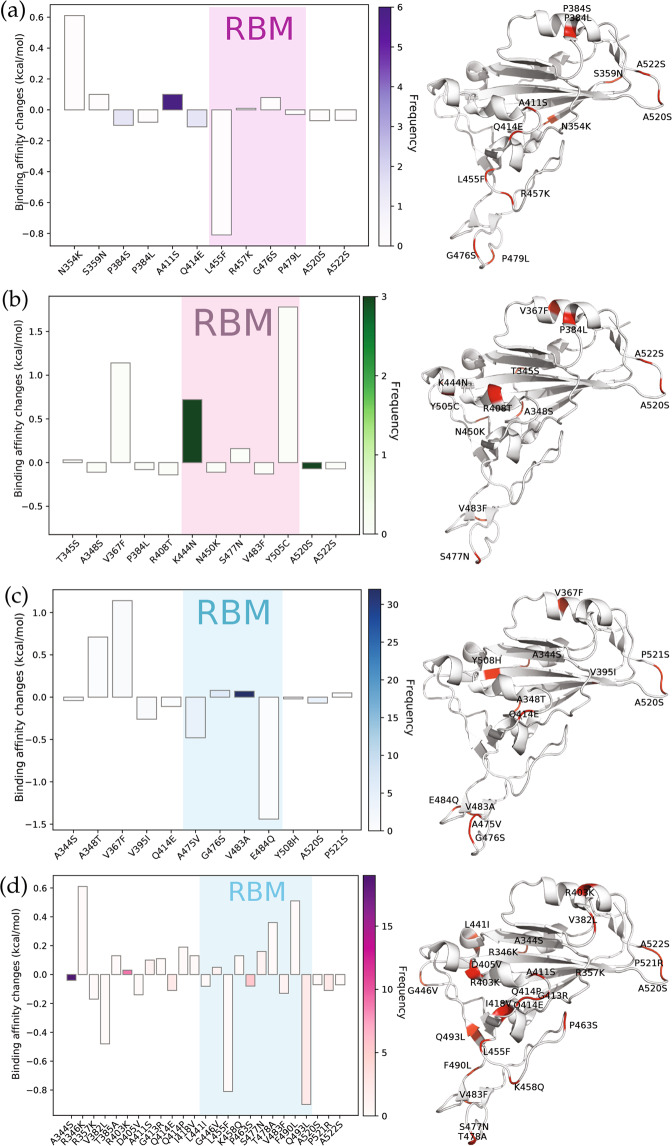
Binding free energy changes ΔΔ*G* (kcal/mol) induced by mutations (figure on the left) and mutations on the SARS-CoV-2 S protein RBD (figure on the right) in four clusters. **a** Cluster A, **b** Cluster B, **c** Cluster C, and **d** Cluster D.

Figure 8a depicts the binding free energy changes of mutations in Cluster A. A total of 12 single mutations are found in Cluster A. Five of them have the positive binding free energy changes, while the other seven mutations induced the negative binding free energy changes. The L455F mutation localized on the RBM has low frequency but the highest absolute binding free energy changes, while the A411S localized outside the RBM with low positive binding free energy change has the highest frequency. Although N354K has relatively high binding free energy change, the frequency is low. Mutations P384S and Q414E have negative binding free energy changes, with the total frequency equals to 4. In general, we can say mutations in Cluster A lead to a less contagious SARS-CoV-2 substrain. It is worth noting that from Table S1, mutations in Cluster A are involved in all of the 20 states with a relatively large number of sequences.

#### Cluster B infectivity

Figure 8b describes the binding free energy changes of mutations in Cluster B. There are 12 single mutations on the S protein RBD and five single mutations on the RBM. Although the number of single mutations with positive binding free energy changes is less than that with negative binding free energy changes, the high frequency of K444N on RBM enhances the infectivity of SARS-CoV-2. Therefore, the mutations in Cluster B may strengthen the infectivity of SARS-CoV-2. We notice that all of the states in Table S1 are associated with Cluster B. Additionally, a large proportion of single mutations in Cluster B are in CA, FL, OR, WA, and WI. In contrast, AZ, DC, and ME only involve fewer than five sequences in Cluster B.

#### Cluster C infectivity

Figure 8c describes the binding free energy changes in Cluster C. Although mutatin A475V on the RBM has a negative binding free energy change with a relatively high frequency (5) on the RBM, the much higher frequencies of two mutations G476S (7) and V483A (31) with positive binding free energy change suggests that the mutations in Cluster C may strengthen the infectivity of SARS-CoV-2 in general. Notably, the V483A mutation is localized on the RBM with the highest frequency, indicating that V483A may favor SARS-CoV-2 by natural selection and cause SARS-CoV-2 more infectious. Although A348T has relatively low frequencies due to the limited number of genome samples, their high binding free energy changes may lead to a more contagious SARS-CoV-2 substrain. It is worth noting that from Table S1, mutations in Cluster C are involved in all of the 20 states except for NM. However, AK, CT, DC, and LA each only have fewer than 5 SARS-CoV-2 isolates related to Cluster C.

#### Cluster D infectivity

The binding free energy changes of RDB mutations in Cluster D are illustrated in Fig. 8d. Twenty-five different single mutations are classified in Cluster D. Among them, 13 mutations have negative binding free energy changes and relatively high frequencies, showing that overall the mutations in Cluster D may reduce the transmission capacity of SARS-CoV-2. Moreover, on the RBM, we can see that mutation Q493L has a relatively high binding free energy change and frequency. In addition, mutation A344S has the highest frequency among the 13 infectivity-weaken mutations. As shown in Table S1, all of these 20 states have mutations in Cluster D, especially in CA, MN, NY, WA, and WI.

Based on the clusters’ infectivity analysis, mutations in Clusters A and D may make the SARS-CoV-2 more contagious, while Clusters B and C may lead to less contagious SARS-CoV-2 strains. However, since the infectivity-strengthening D614G mutation is associated with all clusters and essentially all the US genome isolates, it may be reasonable to say all of the US SARS-CoV-2 substrains become more infectious compared with the original genome collected on 24 December 2019 in China.

## Methods

### Data collection and pre-processing

On 5 January 2020, the complete genome sequence of SARS-CoV-2 was first released on the GenBank (Access number: NC_045512.2)^5^. Since then, there has been a rapid accumulation of SARS-CoV-2 genome sequences. In this work, 45,494 complete genome sequences with high coverage of SARS-CoV-2 strains from the infected individuals in the world were downloaded from the GISAID database^2^ (https://www.gisaid.org/) as of 11 September 2020. All the incomplete records and those without the exact submission date in GISAID were not considered. To rearrange the complete genome sequences according to the reference SARS-CoV-2 genome, multiple sequence alignment (MSA) is carried out by using Clustal Omega^59^ with default parameters.

The amino acid sequence of NSP2, NSP12, NPS13, Spike protein, ORF3a, ORF8, and Nucleocapsid were downloaded from the GenBank^60^. The three-dimensional (3D) structures of NSP12, spike protein, and ORF3a used in this work were extracted from the Protein Data Bank (https://www.rcsb.org/), denoted as 7BTF, 6VYB, and 6XDC, respectively. The 3D structures of NSP2, ORF8, NSP13, and Nucleocapsid were generated by I-TASSER model^61^. The 3D structure graph is created by using PyMOL^39^.

### Single-nucleotide polymorphism calling

Single-nucleotide polymorphism (SNP) calling measures the genetic variations between different members of a species. Establishing the SNP calling method to the investigation of the genotype changes during the transmission and evolution of SARS-CoV-2 is of great importance^9,10^. By analyzing the rearranged genome sequences, SNP profiles, which record all of the SNP positions in teams of the nucleotide changes and their corresponding positions, can be constructed. The SNP profiles of a given SARS-CoV-2 genome isolated from a COVID-19 patient capture all the differences from a complete reference genome sequence and can be considered as the genotype of the individual SARS-CoV-2.

### Distance of SNP variants

In this work, we use the Jaccard distance to measure the similarity between SNP variants and compare the difference between the SNP variant profiles of SARS-CoV-2 genomes.

The Jaccard similarity coefficient is defined as the intersection size divided by the union of two sets *A* and *B*^62^:

1 $$J(A,B)=\frac{| A\cap B| }{| A\cup B| }=\frac{| A\cap B| }{| A| +| B| -| A\cap B| }.$$

The Jaccard distance of two sets *A* and *B* is scored as the difference between one and the Jaccard similarity coefficient and is a metric on the collection of all finite sets:

2 $${d}_{J}(A,B)=1-J(A,B)=\frac{| A\cup B| -| A\cap B| }{| A\cup B| }.$$

Therefore, the genetic distance of two genomes corresponds to the Jaccard distance of their SNP variants.

In principle, the Jaccard distance of SNP variants takes account of the ordering of SNP positions, i.e., transmission trajectory, when an appropriate reference sample is selected. However, one may fail to identify the infection pathways from the mutual Jaccard distances of multiple samples. In this case, the dates of the sample collection provide key information. Additionally, clustering techniques, such as *k*-means, UMAP, and t-distributed stochastic neighbor embedding (t-SNE), enable us to characterize the spread of COVID-19 onto the communities.

### *K*-means clustering

*K*-means clustering aims at partitioning a given data set $X=\{{x}_{1},{x}_{2},\cdots \ ,{x}_{n},\cdots \ ,{x}_{N}\},{x}_{n}\in {{\mathbb{R}}}^{d}$ into *k* clusters {*C*_1_, *C*_2_, ⋯ , *C*_*k*_}, *k* ≤ *N* such that the specific clustering criteria are optimized. The standard *K*-means clustering algorithm picks *k* points as cluster centers randomly at beginning and separates each data to its nearest cluster. Here, *k* cluster centers will be updated subsequently by minimizing the within-cluster sum of squares (WCSS):

3 $$\mathop{\sum }\nolimits_{i = 1}^{k}\sum _{{x}_{i}\in {C}_{k}}\parallel {x}_{i}-{\mu }_{k}{\parallel }_{2}^{2},$$

where *μ*_*k*_ is the mean of points locating in the *k*th cluster *C*_*k*_ and *n*_*k*_ is the number of points in *C*_*k*_. Here, ∥⋅∥_2_ denotes the *L*_2_ distance.

The aforementioned algorithm offers an optimal partition of *k* clusters. However, it is more important to find the best number of clusters for the given set of SNP variants. Therefore, the Elbow method is employed. A set of WCSS can be calculated in the *k*-means clustering process by varying the number of clusters *k*, and then plot WCSS according to the number of clusters. The optimal number of clusters will be the elbow in this plot. The WCSS measures the variability of the points within each cluster which is influenced by the number of points *N*. Therefore, as the number of total points of *N* increases, the value of WCSS becomes larger. Additionally, the performance of *k*-means clustering depends on the selection of the specific distance metric.

In this work, we implement *k*-means clustering with the Elbow method for analyzing the optimal number of the subtypes of SARS-CoV-2 SNP variants. The Jaccard distance-based representation is considered as the input features for the *k*-means clustering method. If we have a total of *N* SNP variants concerning a reference genome in a SARS-CoV-2 sample, the location of the mutation sites for each SNP variant will be saved in the set *S*_*i*_, *i* = 1, 2, ⋯ , *N*. The Jaccard distance between two different sets (or samples) *S*_*i*_, *S*_*j*_ is denoted as *d*_*J*_(*S*_*i*_, *S*_*j*_). Therefore, the *N* × *N* Jaccard distance-based representation is

4 $${D}_{J}(i,j)={d}_{J}({S}_{i},{S}_{j}).$$

This representation is used in our *k*-means clustering.

### Topology-based machine learning prediction of protein–protein binding free energy changes following mutations

The topology-based network tree (TopNetTree) model was developed by an innovative integration between the topological representation and network tree (NetTree) to predict the binding free energy changes of protein–protein interaction (PPI) following mutation ΔΔ*G*^28^. The TopNetTree is applied to predict the binding free energy changes upon mutations that occurred on the RBD of SARS-CoV-2. Algebraic topology^30^ is utilized to simplify the structural complexity of protein–protein complexes and embed vital biological information into topological invariants. NetTree integrates the advantages of convolutional neural networks (CNN) and gradient-boosting trees (GBT), such that CNN is treated as an intermediate model that converts vectorized element- and site-specific persistent homology features into a higher-level abstract feature, and GBT uses the upstream features and other biochemistry features for prediction. The performance test of tenfold cross-validation on the dataset (SKEMPI 2.0^63^) carried out using gradient boosted regression trees (GBRTs). The errors with the SKEMPI2.0 dataset are 0.85 in terms of Pearson correlations coefficient (*R*_*p*_) and 1.11 kcal/mol in terms of the root mean square error (RMSE)^28^.

### Topology-based machine learning prediction of protein folding stability changes following mutation

In this work, the prediction of protein folding stability changes upon mutation is carried out using a topology-based mutation predictor (TML-MP) (https://weilab.math.msu.edu/TML/TML-MP/) which was introduced in literature^27^. The folding stability change following mutation ΔΔ*G* = Δ*G*_w_−Δ*G*_m_ measures the difference between the folding free energies of the wild type Δ*G*_w_ and the mutant type Δ*G*_w_. More specifically, a positive folding stability change ΔΔ*G* indicates that the mutation will stabilize the structure of the protein and vice versa. The essential biological information is revealed by persistent homology^30^. The machine learning features are generated by the element-specific persistent homology and biochemistry techniques. The dataset includes 2648 mutations cases in 131 proteins provided by Dehouck et al.^64^ and is trained by a gradient boosted regression trees (GBRTs). The error with the corresponding dataset is given as Pearson correlations coefficient (*R*_*p*_) of 0.79 and root mean square error (RMSE) of 0.91 kcal/mol from previous work^27^.

The persistent homology is widely applied in a variety of practical feature generation problems^30^. It is also successful in the implementation of predictions of protein folding stability changes upon mutation^27^. The key idea in TML-MP is using the element-specific persistent homology (ESPH) which distinguishes different element types of biomolecules when building persistent homology barcodes. Commonly occurring protein element types include C, N, O, S, and H, where hydrogen and sulfur are excluded according to that hydrogen atoms are often absent from PDB data and sulfur atoms are too few in most proteins to be statistically important. Thus, C, N, and O elements are considered on the ESPH in protein characterization. Features are extracted from the different dimensions of persistent homology barcodes by dividing barcodes into several equally spaced bins which is called binned barcode representation. The auxiliary features, such as geometry, electrostatics, amino acid type composition, and amino acid sequence, are included for machine learning training as well. In TML-MP, gradient boosted regression trees (GBRTs)^29^ are employed to train the dataset according to the size of the training dataset, absence of model overfitting, non-normalization of features, and ability of nonlinear properties ^27^.

### Graph network models

Graph networks can model interactions and their strength between pairs of units in molecules. These approaches are employed to understand mutation-induced structural changes. The biological and chemical properties are measured by comparing descriptors on different networks. In this work, the network consists of a set *S* of C_*α*_ atoms from every residue of protein structure except the target mutation residue such that a C_*α*_ atom is included if it is within 16 Å to any atom of the target mutation. The total atom set *T* is defined as the atoms (C, N, and O) of the target residue and C_*α*_ atoms of the network set *S*. Moreover, two vertices are connected in the network if their distance is <8 Å. Thus the adjacency matrix *A* can be defined as well where *A* is a matrix containing 0 and 1 such that *A*(*i*, *j*) = 0 if *i*th and *j*th atoms are disconnected and *A*(*i*, *j*) = 1 if *i*th and *j*th atoms are connected. Two graph network models employed in this work are described below.

#### Flexibility-rigidity index

FRI was introduced to study the flexibility of protein molecules^25,26^. The single residue molecular rigidity index measures its influence on the set *S* which is given as

5 $${R}_{\eta }^{\alpha }=\mathop{\sum }\limits_{i=1}^{{N}_{S}}\mathop{\sum }\limits_{j=1}^{{N}_{T}}{e}^{-\left(\right.\frac{\parallel {{\bf{r}}}_{i}-{{\bf{r}}}_{j}\parallel }{\eta }{\left)\right.}^{2}},$$

where *α* = w or m stands for the wild type w or the mutant type m, *N*_*S*_ is the number of C_*α*_ atoms of the set *S*, and *N*_*T*_ is the number of atoms in total atom set *T*. Here, ∥**r**_*i*_ − **r**_*j*_∥ is the distance between atoms at **r**_*i*_ and **r**_*j*_.

The molecular FRI rigidity *R*_*η*_ measures the topological connectivity and the geometric compactness of the network consisting of C_*α*_ at each residue and the heavy atoms involved in the mutant.

#### Average subgraph centrality

Average subgraph centrality is built on the exponential of the adjacency matrix, *E* = *e*^*A*^, where *A* is the aforementioned adjacency matrix. The subgraph centrality is the summation of weighted closed walks of all lengths starting and ending at the same node^11,31^. Thus the average subgraph centrality reveals the average of participating rate of each vertex in all subgraph and the network motif, which is given as

6 $$\langle {C}_{s}^{\alpha }\rangle =\frac{1}{{N}_{S}}\mathop{\sum }\limits_{i=1,i\notin {I}_{T}}^{N}E(i,i),$$

where *I*_*T*_ is the index set of the mutation residue.

### Reporting summary

Further information on research design is available in the Nature Research Reporting Summary linked to this article.

## Supplementary information

Supplementary Information

Description of Additional Supplementary Files

Supplementary Data

Reporting Summary

## Data Availability

In this work, 45,494 complete genome sequences with high coverage of SARS-CoV-2 strains from the infected individuals in the world were downloaded from the GISAID database^2^ (https://www.gisaid.org/) as of 11 September 2020. The reference genome of SARS-CoV-2 was first released on the GenBank (Access number: NC_045512.2)^5^. All of the complete genome sequences we used in this paper are listed in the Section 2 of the Supplementary Information. Supplementary Data 1 lists the GISAID IDs we use in the world. Supplementary Data 2 lists the GISAID IDs we use in the Untied States. Supplementary Data 3 contains the world clusters information and Supplementary Data 4 has the US clusters information. Supplementary Data 5–13 are the acknowlegement table provided by GISAID. The amino acid sequence of NSP2, NSP12, NPS13, Spike protein, ORF3a, ORF8, and Nucleocapsid were downloaded from the GenBan. The three-dimensional (3D) structures of NSP12, spike protein, and ORF3a used in this work were extracted from the Protein Data Bank (https://www.rcsb.org/), denoted as 7BTF, 6VYB, and 6XDC, respectively. The 3D structures of NSP2, ORF8, NSP13, and Nucleocapsid were generated by I-TASSER model^61^. The 3D structure graph is created by using PyMOL^39^.

## Code availability

We use the Cluster Omega (https://www.ebi.ac.uk/Tools/msa/clustalo) to do the multiple sequence alignment. The K-mean clustering is emloyed by an open-sopurce package (https://scikit-learn.org/stable/modules/generated/sklearn.cluster.KMeans.html). The code of topology-based machine learning prediction of protein-protein bidning affinity changes following mutations is avaiable at the online server reported in (10.1038/s42256-020-0149-6).
