# Supplementary material for: Analysis of SARS-CoV-2 mutations in the United States suggests presence of four substrains and novel variants
Source: Commun Biol. 2021 Feb 15;4:228. doi: 10.1038/s42003-021-01754-6 (PMC7884689; doi:10.1038/s42003-021-01754-6)
Supplement: Supplementary file 1 — Supplementary Information [file 42003_2021_1754_MOESM1_ESM.pdf]

Supplementary Information
Analysis of SARS-CoV-2 mutations in the United States suggests presence of four substrains and novel variants

Rui Wang[1], Jiahui Chen[1], Kaifu Gao[1], Yuta Hozumi [1], Changchuan Yin[2], and Guo-Wei Wei[1,3,4*]
[1] Department of Mathematics,
Michigan State University, MI 48824, USA.
[2] Department of Mathematics, Statistics, and Computer Science,
University of Illinois at Chicago, Chicago, IL 60607, USA
[3] Department of Electrical and Computer Engineering,
Michigan State University, MI 48824, USA.
[4] Department of Biochemistry and Molecular Biology,
Michigan State University, MI 48824, USA.

October 17, 2020

Contents

S1 Supplementary Tables **1**

S2 Supplementary Data **1**

S3 Supplementary Figures **2**
S3.1 Mutation Tracker 2
S3.2 K-Means clustering 2
S3.3 Proteoforms 3
S3.4 Protein structures, network analysis, and alignment 4

*Corresponding author. E-mail: weig@msu.edu

S1 Supplementary Tables

Table S1 shows the cluster distributions of samples (N_{NS}), total SNP counts (N_{TF}), and the ratios of SNPs (R_{TF} = $N_{\mathrm{TF}}/7123$) in 20 states of the U.S. The 20 states are Alaska (AK), Arizona (AZ), California (CA), Connecticut (CT), Washington, D.C. (DC), Florida(FL), Louisiana (LA), Maine (ME), Maryland (MD), Michigan (MI), Minnesota (MN), Nevada(NV), New Mexico (NM), New York (NY), Oregon (OR), Texas(TX), Utah (UT), Virginia (VA), Washington (WA), and Wisconsin (WI).

Table S2 lists the number of samples (N_{NS}) of a particular mutation on 5'UTR in the United States and worldwide. Here, only the mutations that occurred more than 9 times are listed.

Table S1: The cluster distributions of samples (N_{NS}), total SNP counts (N_{TF}), and the ratios of SNPs (R_{TF} = $N_{\mathrm{TF}}/7123$) in 20 states of the U.S. The 20 states are Alaska (AK), Arizona (AZ), California (CA), Connecticut (CT), Washington, D.C. (DC), Florida(FL), Louisiana (LA), Maine (ME), Maryland (MD), Michigan (MI), Minnesota (MN), Nevada(NV), New Mexico (NM), New York (NY), Oregon (OR), Texas(TX), Utah (UT), Virginia (VA), Washington (WA), and Wisconsin (WI).

	Cluster A			Cluster B			Cluster C			Cluster D		
State	N_{NS}	N_{TF}	R_{TF}	N_{NS}	N_{TF}	R_{TF}	N_{NS}	N_{TF}	R_{TF}	N_{NS}	N_{TF}	R_{TF}
AK	13	108	1.5%	15	183	2.6%	4	27	0.4%	19	154	2.2%
AZ	36	273	3.8%	4	34	0.5%	12	95	1.3%	25	224	3.1%
CA	461	3574	50.2%	544	6147	86.3%	258	1507	21.2%	566	5440	76.4%
CT	29	226	3.2%	17	166	2.3%	3	35	0.5%	67	756	10.6%
DC	13	94	1.3%	2	54	0.8%	4	29	0.4%	4	36	0.5%
FL	122	907	12.7%	132	1531	21.5%	47	522	7.3%	299	3492	49.0%
LA	179	1451	20.4%	7	188	2.6%	1	15	0.2%	163	1753	24.6%
ME	26	175	2.5%	4	31	0.4%	13	101	1.4%	47	304	4.3%
MD	45	359	5.0%	17	320	4.5%	29	365	5.1%	78	899	12.6%
MI	253	1868	26.2%	10	100	1.4%	16	157	2.2%	90	900	12.6%
MN	209	1590	22.3%	52	622	8.7%	159	1260	17.7%	312	3576	50.2%
NV	52	395	5.5%	8	79	1.1%	19	107	1.5%	69	728	10.2%
NM	17	115	1.6%	15	138	1.9%	0	0	0.0%	8	71	1.0%
NY	925	6700	94.1%	68	651	9.1%	96	675	9.5%	300	2846	40.0%
OR	41	337	4.7%	153	1753	24.6%	70	401	5.6%	255	2503	35.1%
TX	10	79	1.1%	9	114	1.6%	8	95	1.3%	15	160	2.2%
UT	39	293	4.1%	49	505	7.1%	21	159	2.2%	252	2819	39.6%
VA	199	1561	21.9%	63	803	11.3%	35	274	3.8%	291	3146	44.2%
WA	595	4593	64.5%	1213	15630	219.4%	1155	9005	126.4%	680	7925	111.3%
WI	111	806	11.3%	322	4219	59.2%	187	934	13.1%	414	4324	60.7%

Table S2: The number of samples (N_{NS}) of a particular mutation on 5'UTR in the United States and worldwide. Here, only the mutations that occurred more than 9 times are listed.

Mutation	US N_{NS}	World N_{NS}	Mutation	US N_{NS}	World N_{NS}
241C>T	9628	36786	187A>G	36	207
199G>T	28	33	222C>T	17	50
208G>T	13	15	218C>T	13	29
242G>T	11	17	169A>G	10	11

S2 Supplementary Data

Total 13 spreadsheets are merged in the Supplementary Data.xlsx:

Supplementary Data 1: The GISAID IDs we use in the world. (Up to September 11, 2020).

Supplementary Data 2: The GISAID IDs we use in the United States. (Up to September 11, 2020).

Supplementary Data 3: The world clusters information.

Supplementary Data 4: The US clusters information.

Supplementary Data 5: Acknowledgment table provided by GISAID in Jan 2020.

Supplementary Data 6: Acknowledgment table provided by GISAID in Feb 2020.

Supplementary Data 7: Acknowledgment table provided by GISAID in March 2020.

Supplementary Data 8: Acknowledgment table provided by GISAID in April 2020.

Supplementary Data 9: Acknowledgment table provided by GISAID in May 2020.

Supplementary Data 10: Acknowledgment table provided by GISAID in June 2020.

Supplementary Data 11: Acknowledgment table provided by GISAID in July 2020.

Supplementary Data 12: Acknowledgment table provided by GISAID in August 2020.

Supplementary Data 13: Acknowledgment table provided by GISAID in September 2020.

S3 Supplementary Figures

S3.1 Mutation Tracker

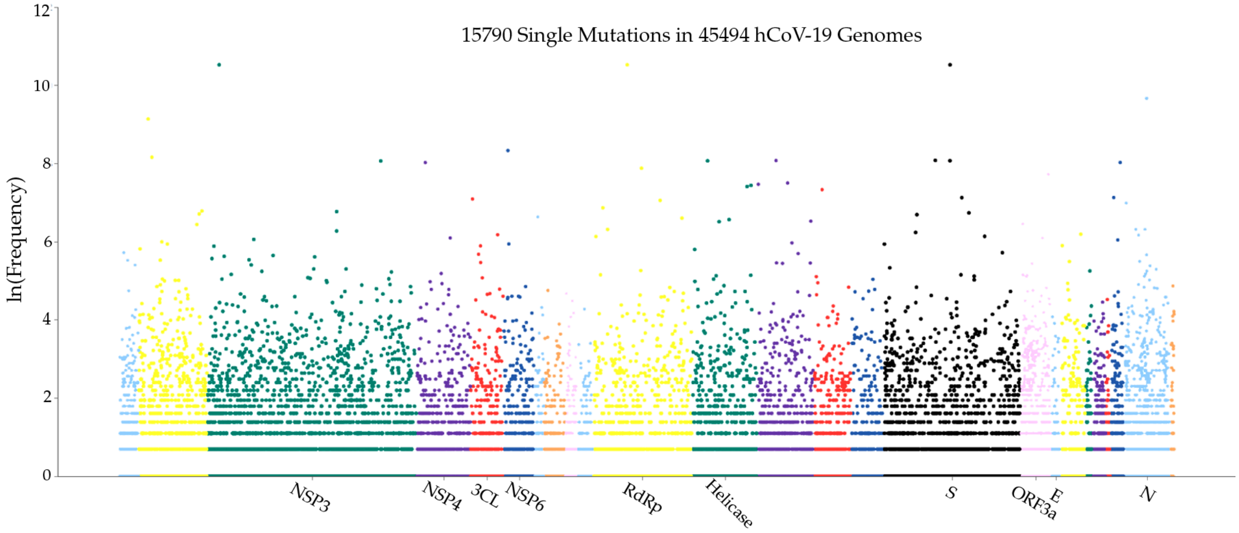

Figure S1: Genome-wide SARS-CoV-2 mutation distribution. The y-axis represents the natural log frequencies of mutations on specific positions of the complete SARS-CoV-2 genome. A few landmark positions are labeled with gene (protein) names.

S3.2 K-Means clustering

The k-means clustering is used to classify the SARS-CoV-2 the SNP variants. The Elbow method is used to determine the optimal number of clusters. Our results demonstrate four main clusters in the United States (US) as shown in Figure S2, which plots the within-cluster sum of squares according to the number

of clusters k for the SNP variants in the United States based on Jaccard distance metric. The optimal values of k-mean clusters is shown as the turning point in the in the elbow plots.

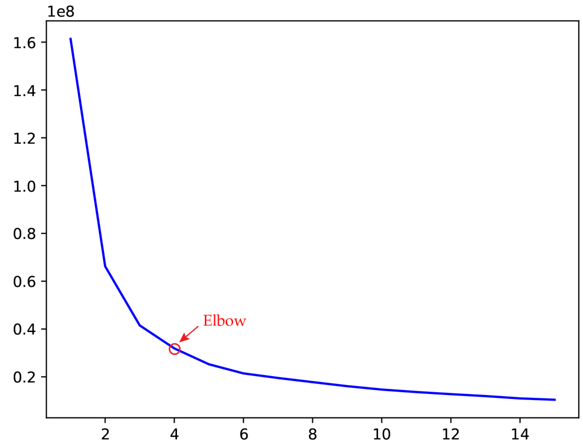

Figure S2: The plot of WCSS according to the number of clusters based on Jaccard distance metric. Here, Jaccard distance-based representation is taken as the input feature. The arrows point out the optimal number of clusters. The within-cluster sum of squares against the number of clusters for the SNP variants in the United States. The optimal number of clusters in the United States is four.

S3.3 Proteoforms

Figure S3 shows the visualization of the proteoforms of SARS-CoV-2 NSP2, NSP13, NSP12, spike protein, ORF8, and ORF3a. The top 8 mutations are marked in coor. The red color represents the wild type residue and the yellow color represents the mutant type.

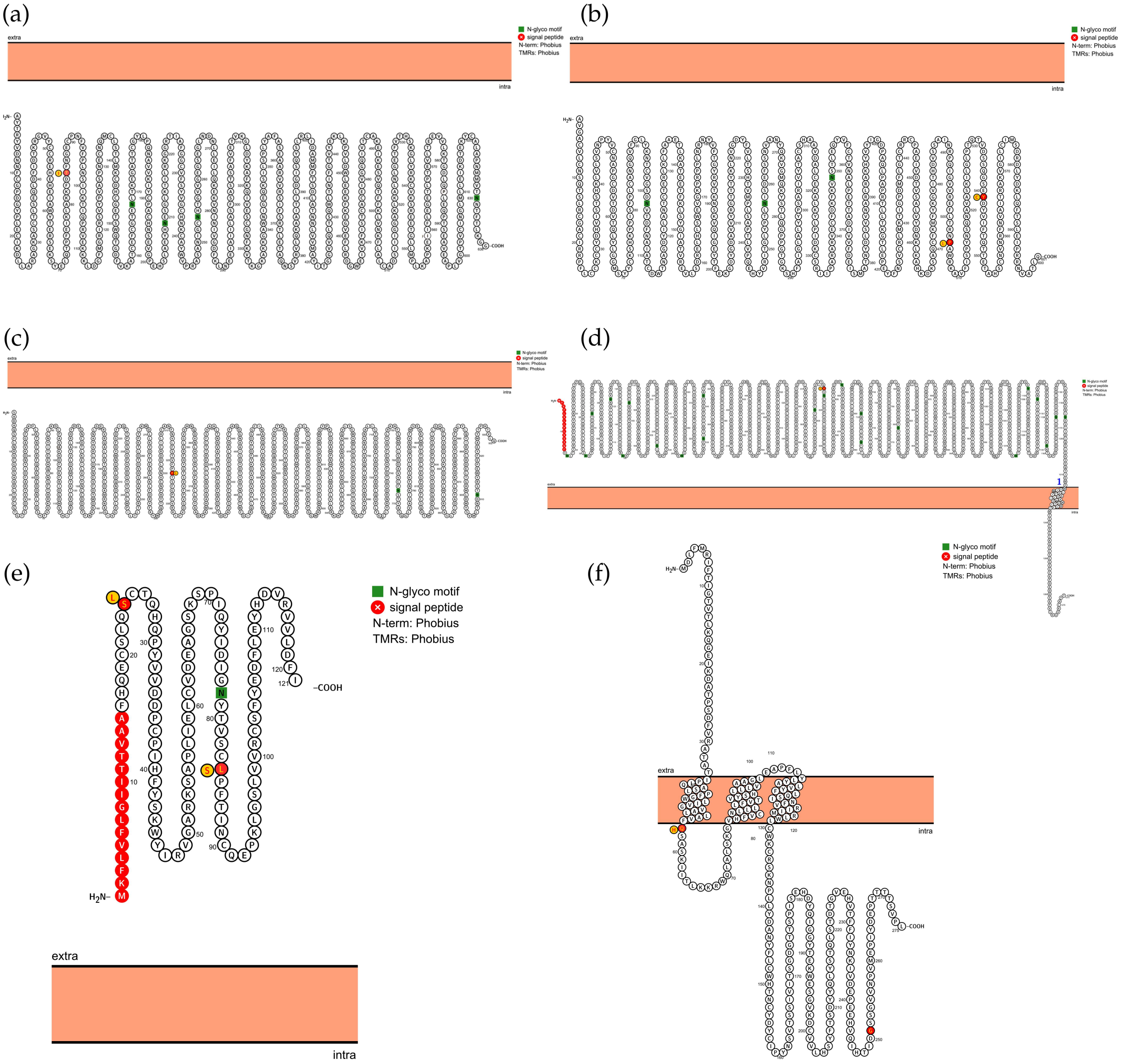

Figure S3: The visualization of six SARS-CoV-2 proteoforms. (a) Proteoform of NSP2. (b) Proteoform of NSP13. (c) Proteoform of NSP12. (d) Proteoform of spike protein. (e) Proteoform of ORF8. (f) Proteoform of ORF3a. The red color represents the wild type and the yellow represents the wild type.

S3.4 Protein structures, network analysis, and alignment

Figure S6 illustrates the 3D structure of SARS-CoV-2 NSP2 protein and the difference of FRI rigidity index, the subgraph centrality between the network with wild type and the network with mutant type.

Figure S7 illustrates sequence alignments for the NSP2 of SARS-CoV-2, SARS-CoV, bat coronavirus RaTG13, bat coronavirus CoVZC45, bat coronavirus BM48-31. Detailed numbering is given according to SARS-CoV-2. One high-frequency mutation 1059C>T-(T85I) locates on the NSP2 protein. Here, the red rectangle marks the T85I position with its neighborhoods.

Figure S8 illustrates the sequence alignments for the NSP13 protein of SARS-CoV-2, SARS-CoV, bat coronavirus RaTG13, bat coronavirus CoVZC45, bat coronavirus BM48-31. Detailed numbering is given according to SARS-CoV-2. Two high-frequency mutations 17858A>G-(Y541C) and 17747C>T-(P504L) locate on NSP13. Here, the red rectangles mark the Y541C and P504: mutations with their neighborhoods.

Figure S9 illustrate the 3D structure of SARS-CoV-2 NSP13 protein and the difference of FRI rigidity index, the subgraph centrality between the network with wild type and the network with mutant type.

Figure S10 illustrates the sequence alignments for the ORF8 protein of SARS-CoV-2, SARS-CoV, bat coronavirus RaTG13, bat coronavirus CoVZC45, bat coronavirus BM48-31. Detailed numbering is given according to SARS-CoV-2. Two high-frequency mutations 28144T>C-(L84S) and 27964C>T-(S24L) locate on the ORF8. Here, the red rectangles mark the S24L and L84S mutations with their neighborhoods.

Figure S11 illustrate the 3D structure of SARS-CoV-2 ORF8 protein and the difference of FRI rigidity index, the subgraph centrality between the network with wild type and the network with mutant type.

Figure S12 illustrate the 3D structure of SARS-CoV-2 N protein and the difference of FRI rigidity index, the subgraph centrality between the network with wild type and the network with mutant type.

Figure S13 illustrates the sequence alignments for the N protein of SARS-CoV-2, SARS-CoV, bat coronavirus RaTG13, bat coronavirus CoVZC45, bat coronavirus BM48-31. Detailed numbering is given according to SARS-CoV-2. Three high-frequency mutations 28881G>A-(R203K), 28882G>A-(R203K), and 28883G>C-(G204R) locate on the N protein. Here, the red rectangles mark mutations R203K and G204R with their neighborhoods.

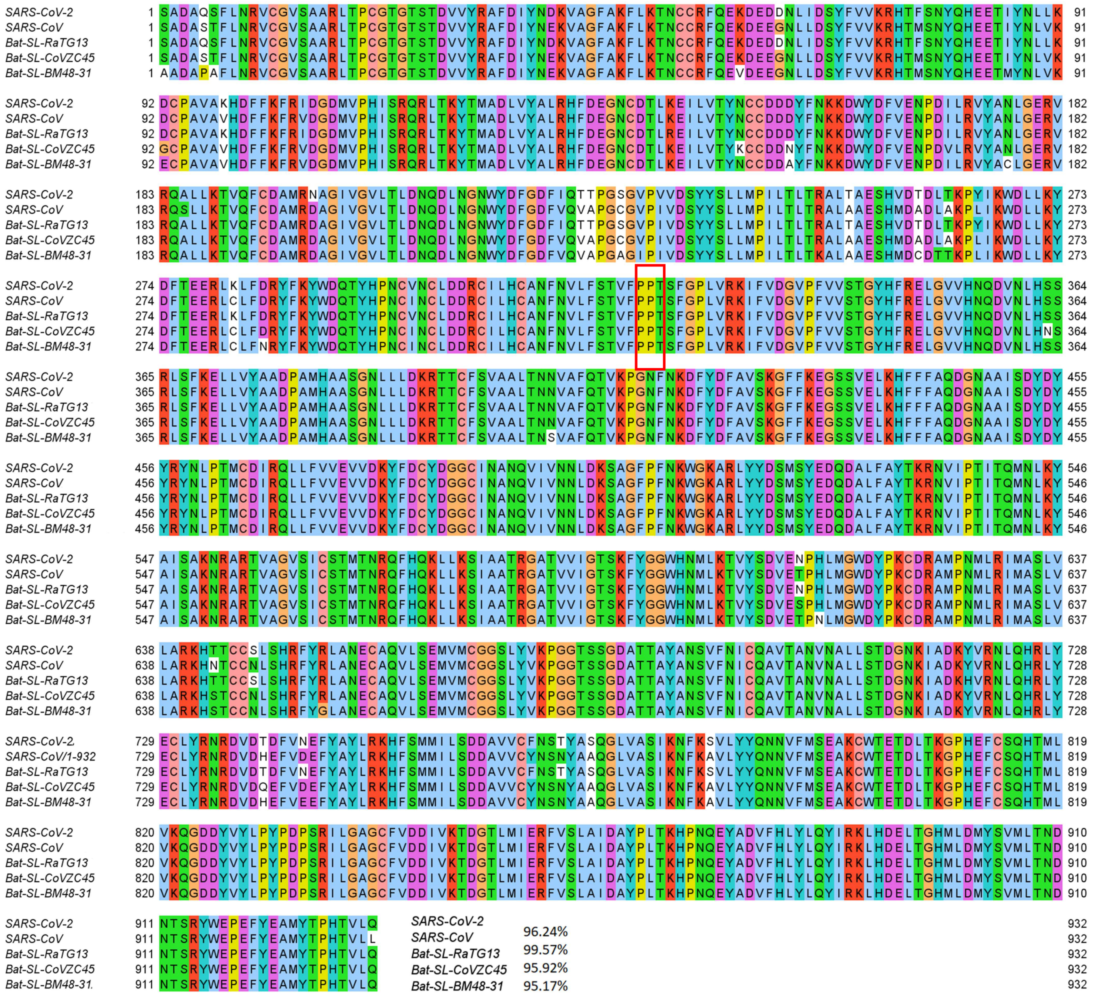

Figure S4: Sequence alignments for the NSP12 of SARS-CoV-2, SARS-CoV, bat coronavirus RaTG13, bat coronavirus CoVZC45, bat coronavirus BM48-31. Detailed numbering is given according to SARS-CoV-2. One high-frequency mutation 14408C>T-(P323L) is detected on the NSP12 protein. Here, the red rectangle marks the P323L mutations with its neighborhoods.

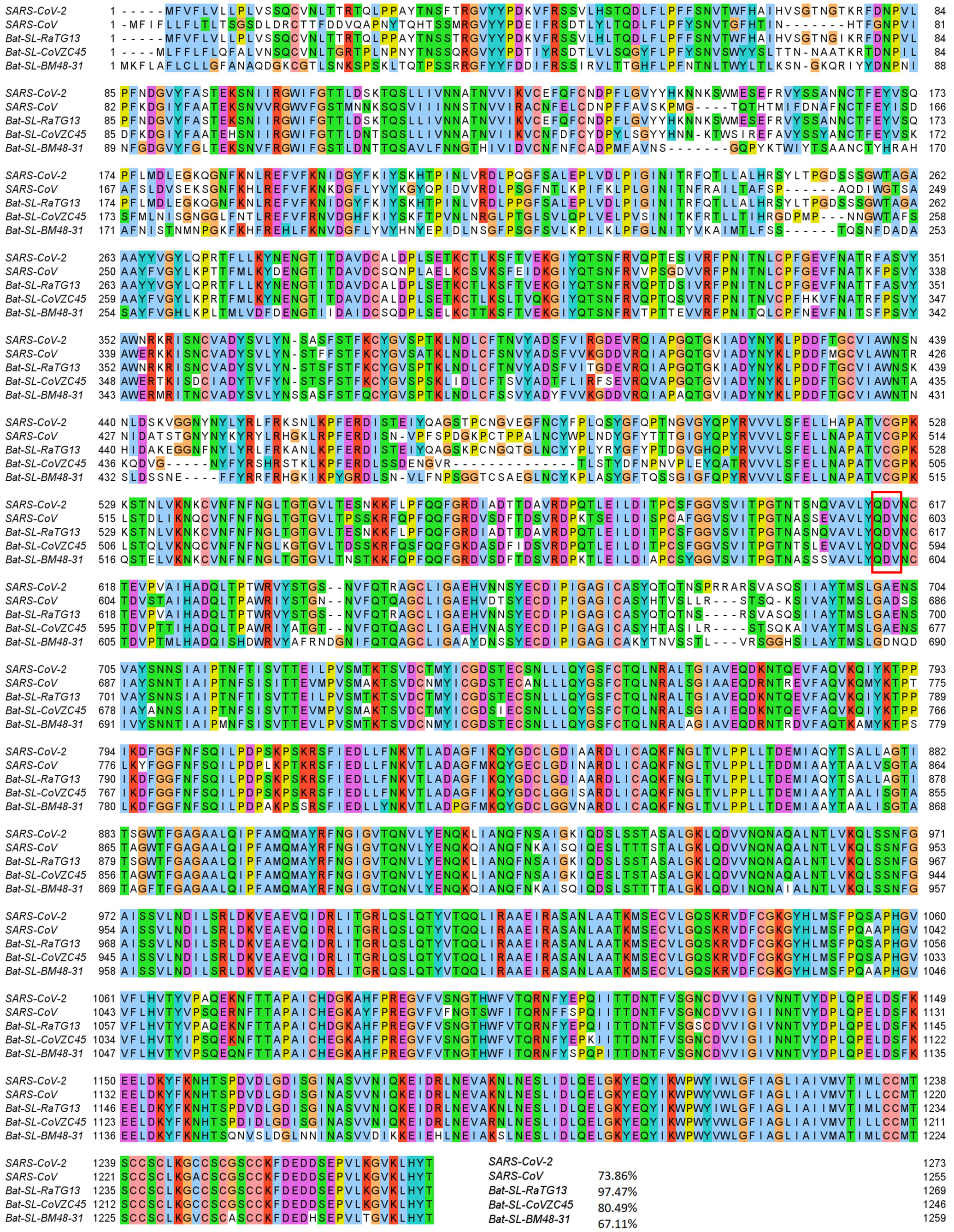

Figure S5: Sequence alignments for the S proteins of SARS-CoV-2, SARS-CoV, bat coronavirus RaTG13, bat coronavirus CoVZC45, bat coronavirus BM48-31. Detailed numbering is given according to SARS-CoV-2 S protein. One high-frequency mutation 23403A>G-(D614G) is detected on the S protein. Here, the red rectangle marks the D614G mutations with its neighborhoods

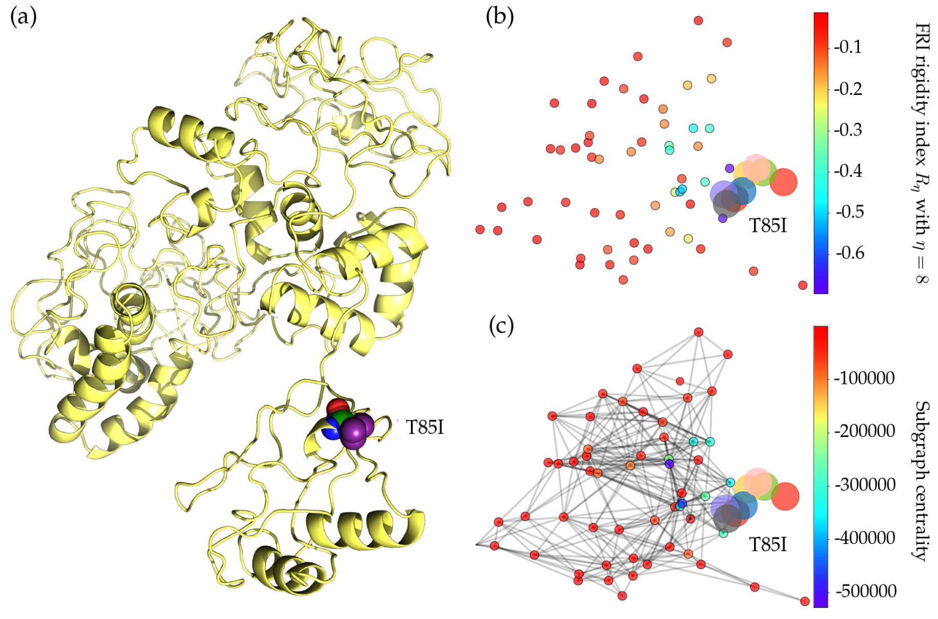

Figure S6: (a) The 3D structure of SARS-CoV-2 NSP2 protein. The mutant residue is marked with color balls. (b) The difference of FRI rigidity index between the network with wild type and the network with mutant type. (c) The difference of the subgraph centrality between the network with wild type and the network with mutant type.

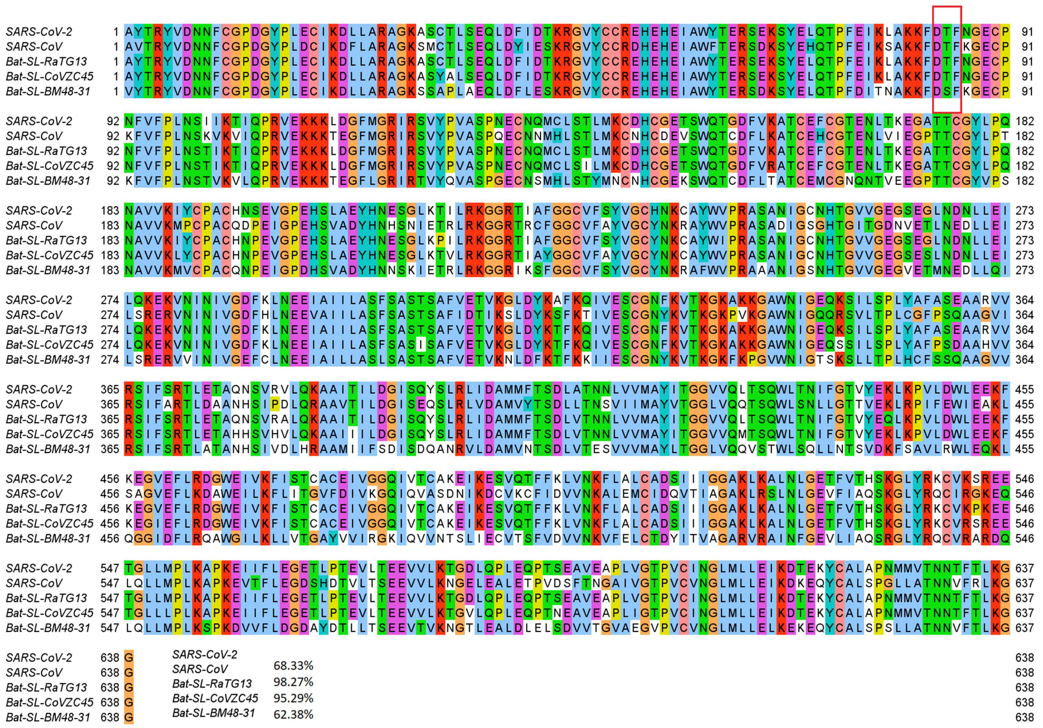

Figure S7: Sequence alignments for the NSP2 of SARS-CoV-2, SARS-CoV, bat coronavirus RaTG13, bat coronavirus CoVZC45, bat coronavirus BM48-31. Detailed numbering is given according to SARS-CoV-2. One high-frequency mutation 1059C>T-(T85I) locates on the NSP2 protein. Here, the red rectangle marks the T85I position with its neighborhoods.

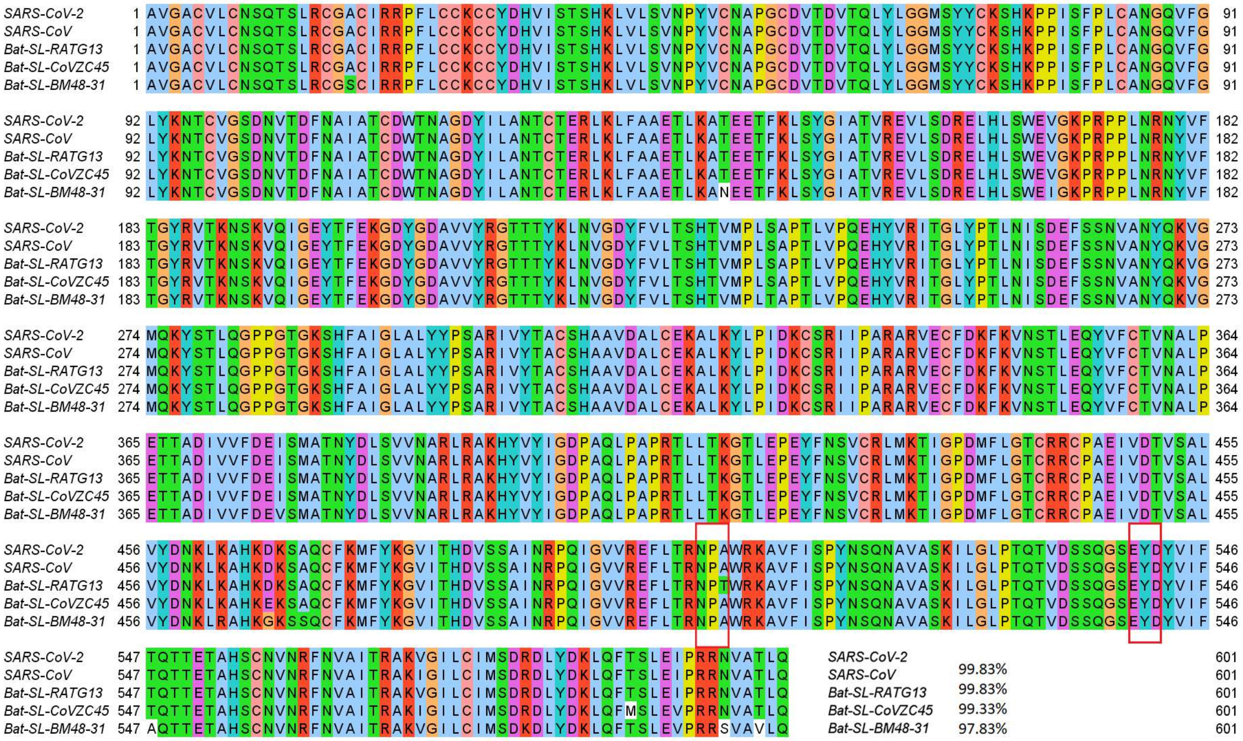

Figure S8: Sequence alignments for the NSP13 protein of SARS-CoV-2, SARS-CoV, bat coronavirus RaTG13, bat coronavirus CoVZC45, bat coronavirus BM48-31. Detailed numbering is given according to SARS-CoV-2. Two high-frequency mutations 17858A>G-(Y541C) and 17747C>T-(P504L) locate on NSP13. Here, the red rectangles mark the Y541C and P504: mutations with their neighborhoods.

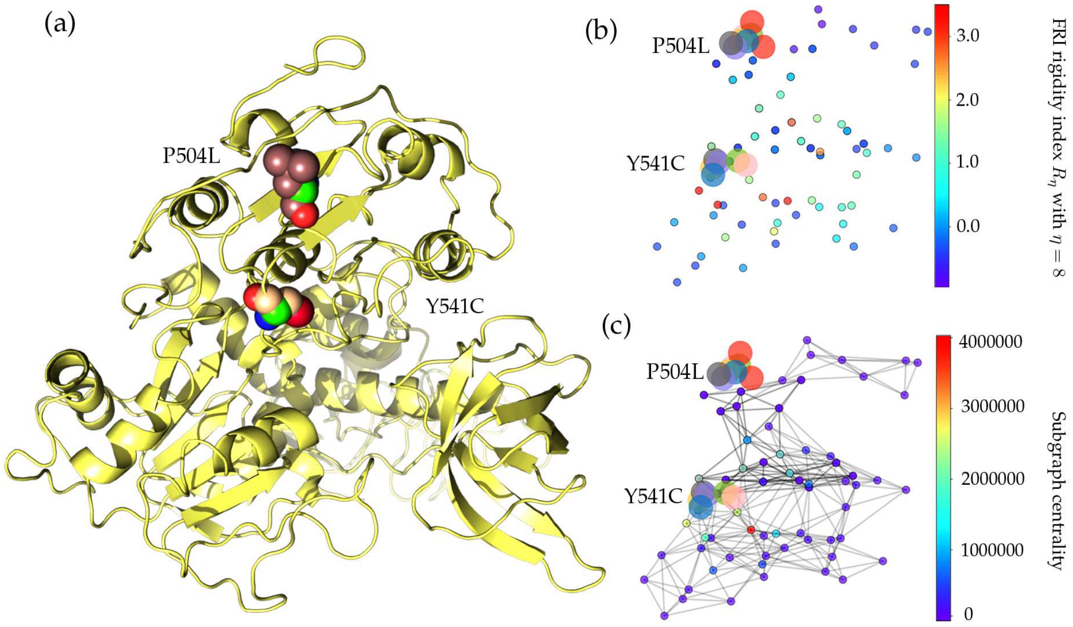

Figure S9: (a) The 3D structure of SARS-CoV-2 NSP13 protein. The mutant residue is marked with color balls. (b) The difference of FRI rigidity index between the network with wild type and the network with mutant type. (c) The difference of the subgraph centrality between the network with wild type and the network with mutant type.

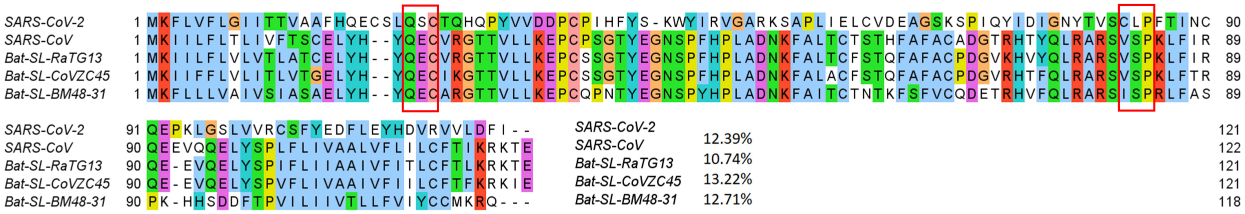

Figure S10: Sequence alignments for the ORF8 protein of SARS-CoV-2, SARS-CoV, bat coronavirus RaTG13, bat coronavirus CoVZC45, bat coronavirus BM48-31. Detailed numbering is given according to SARS-CoV-2. Two high-frequency mutations 28144T>C-(L84S) and 27964C>T-(S24L) locate on the ORF8. Here, the red rectangles mark the S24L and L84S mutations with their neighborhoods.

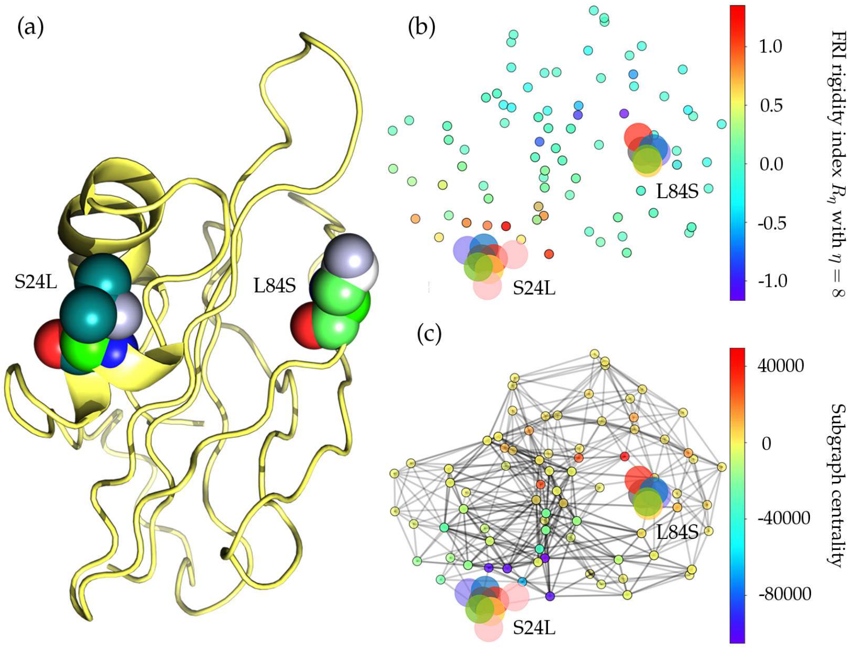

Figure S11: (a) The 3D structure of SARS-CoV-2 ORF8 protein. The mutant residue is marked with color balls. (b) The difference of FRI rigidity index between the network with wild type and the network with mutant type. (c) The difference of the subgraph centrality between the network with wild type and the network with mutant type.

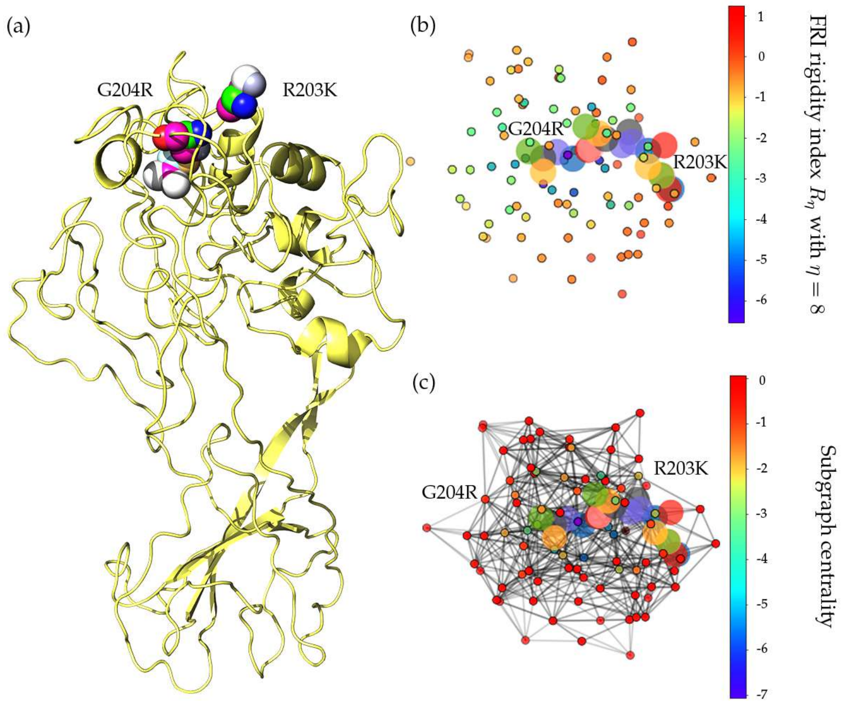

Figure S12: (a) The 3D structure of SARS-CoV-2 N protein. The mutant residue is marked with color balls. (b) The difference of FRI rigidity index between the network with wild type and the network with mutant type. (c) The difference of the subgraph centrality between the network with wild type and the network with mutant type.

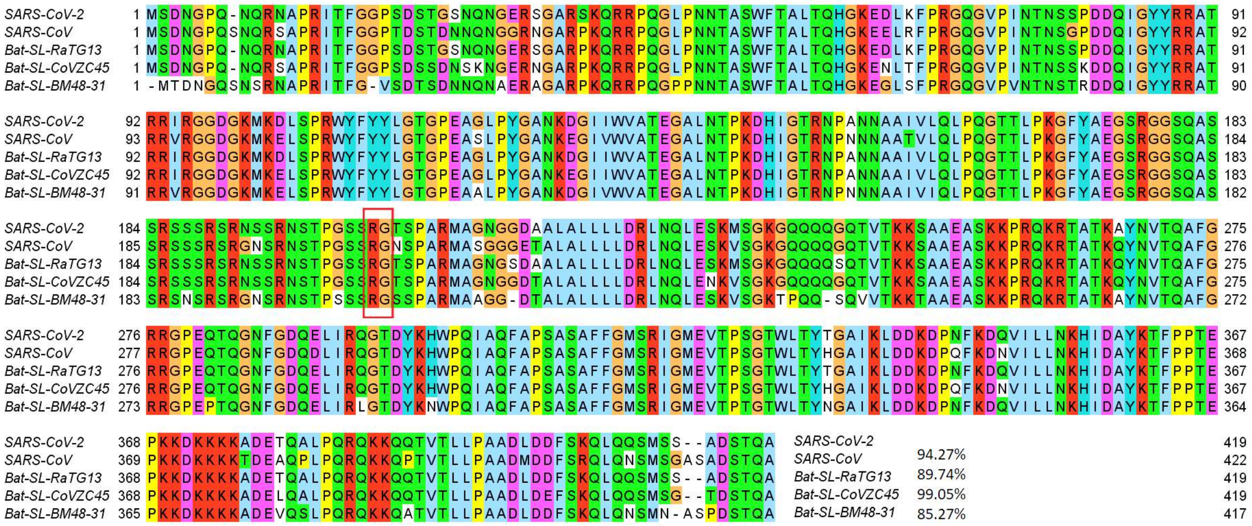

Figure S13: Sequence alignments for the N protein of SARS-CoV-2, SARS-CoV, bat coronavirus RaTG13, bat coronavirus CoVZC45, bat coronavirus BM48-31. Detailed numbering is given according to SARS-CoV-2. Three high-frequency mutations 28881G>A-(R203K), 28882G>A-(R203K), and 28883G>C-(G204R) locate on the N protein. Here, the red rectangles mark mutations R203K and G204R with their neighborhoods.
