# Supplementary material for: Analysis of SARS-CoV-2 mutations in the United States suggests presence of four substrains and novel variants
Source: Commun Biol. 2021 Feb 15;4:228. doi: 10.1038/s42003-021-01754-6 (PMC7884689; doi:10.1038/s42003-021-01754-6)
Supplement: Supplementary file 2 — Description of Additional Supplementary Files [file 42003_2021_1754_MOESM2_ESM.pdf]

Description of Additional Supplementary Files

File name: Supplementary Data

Description:

Supplementary Data 1: The GISAID IDs we use in the world. (Up to September 11, 2020).

Supplementary Data 2: The GISAID IDs we use in the United States. (Up to September 11, 2020).

Supplementary Data 3: The world clusters information.

Supplementary Data 4: The US clusters information.

Supplementary Data 5: Acknowledgment table provided by GISAID in Jan 2020.

Supplementary Data 6: Acknowledgment table provided by GISAID in Feb 2020.

Supplementary Data 7: Acknowledgment table provided by GISAID in March 2020.

Supplementary Data 8: Acknowledgment table provided by GISAID in April 2020.

Supplementary Data 9: Acknowledgment table provided by GISAID in May 2020.

Supplementary Data 10: Acknowledgment table provided by GISAID in June 2020.

Supplementary Data 11: Acknowledgment table provided by GISAID in July 2020.

Supplementary Data 12: Acknowledgment table provided by GISAID in August 2020.

Supplementary Data 13: Acknowledgment table provided by GISAID in September 2020.
